# Wheat straw increases the defense response and resistance of watermelon monoculture to Fusarium wilt

**DOI:** 10.1186/s12870-019-2134-y

**Published:** 2019-12-11

**Authors:** Lili Tang, Shaorui Nie, Wenhui Li, Chao Fan, Siqi Wang, Fengzhi Wu, Kai Pan

**Affiliations:** 1 College of Horticulture and Landscape Architecture, Northeast Agricultural University, Harbin, Heilongjiang 150030 People’s Republic of China; 2 Institute of Cash Crops, Heilongjiang Academy of Agricultural Sciences, Harbin, 150086 Heilongjiang China; 3 Institute of Crop Cultivation and Tillage, Heilongjiang Academy of Agricultural Sciences, Harbin, 150086 Heilongjiang China

**Keywords:** RNA-Seq, Wheat straw, Lignin, Auxin, Fusarium wilt, Watermelon, *Fusarium oxysporum* f.sp*. niveum*

## Abstract

**Background:**

Wheat straw is a rich resource worldwide. Straw return is an effective strategy to alleviate soil-borne diseases on monoculture watermelon. Previous studies focus on soil structure, physical and chemical properties; however, little is known about the molecular responses on host plant.

**Results:**

No significant difference on the population of *Fusarium oxysporum* f.sp*. niveum* race 1(Fon1) in rhizosphere soil was found between control (no addition of wheat straw) and the treated groups (addition of 1% (T1) or 2% (T2) wheat straw). RNA-Seq analysis showed that 3419 differentially expressed genes were clustered into 8 profiles. KEGG analysis revealed that phenylpropanoid biosynthesis and plant hormone signal transduction were involved in wheat straw induced response in monoculture watermelon. Genes in lignin biosynthesis were found to be upregulated, and the lignin and auxin contents were higher in T1 and T2 compared to the control. Lignin was also enriched and the Fon1 population decreased in watermelon roots treated with wheat straw. The enzyme activities of phenylalanine ammonia-lyase and peroxidase were increased.

**Conclusions:**

Our data suggest that the addition of wheat straw enhances the defense response to Fon1 infection in watermelon through increasing lignin and auxin biosynthesis.

## Background

The watermelon (*Citrullus lanatus* (Thunb.) Matsum. & Nakai var. lanatus) is a major economical crop worldwide. According the Food and Agriculture Organization of the United Nations, about 117 million tons of watermelon were harvested in 2016 (http://www.fao.org/faostat/en). In recent years, however, long-term monoculture has led to widespread of Fusarium wilt [1], which is primarily caused by *Fusarium oxysporum*, a soil-borne fungus that causes approximately 30–50% watermelon yield losses worldwide [2]. Fusarium wilt has previously been controlled mainly by soil fumigation [3], fungicides [4], and the use of resistant cultivars [5]. However, these control measures are not desirable and can directly increase environmental pollution. Recently, biological control, which uses natural antagonists, instead of chemicals, to reduce pest populations, has become a popular alternative for plant disease control [6, 7].

Crop straw is used extensively in modern agriculture worldwide and is also the oldest and most economical management practice to relieve monoculture problems and increase crop yields and quality. It has been widely observed that crop straw is beneficial for reducing soil-borne pathogens. Previous studies showed that root and stem rot of cucumber could be controlled by lettuce incorporation into the soil; grape residue and garlic straw were proven inhibiting Fusarium wilt and root-knot nematodes in tomato, respectively [8–10].

According to the plant-microbe interaction principles, there are two ways to suppressing soil-borne diseases: to enhance pathogen resistance of the host, or reduce the pathogen attack [11, 12]. Therefore, the analysis of the response on gene expression and physiology of the watermelon was important to perform. Different defense mechanisms have been developed in plants to protect themselves against microbial infections. Some plants have physical barriers to prevent the entry of potential pathogens [13]; and some produce plant hormones and defense-related proteins in response to infection [14]. Previous reports showed significant increases of pathogenesis-related proteins, defense enzymes and lignin synthesis in host plants during intercropping and/or companion cropping compared to monoculture [15–17]. However, little is known about the mechanism on how crop straw addition reinforces the resistance to soil-borne disease by host plants in straw-return systems.

In recent years, transcriptional and physiological conject analysis has been used broadly to fathom pathophysiologic and genes changes in response to pathogen infections. For many plants, RNA-Seq transcriptome profiling analyses were a breakthrough point for understanding mechanism of plant disease resistance. Transcriptional and physiological analyses revealed that lignin metabolism was involved in *Botrytis-*induced response in tomato [18]. RNA-Seq analysis elucidated the molecular mechanisms in the resistance response to *Ralstonia solanacearum* in tomato [19]. RNA-Seq analysis also revealed that many photosynthesis-related genes were differentially transcribed in the presence of pathogen in both chrysanthemum and mango [20, 21].

To date, little is known regarding the molecular mechanism on wheat straw induced disease resistance in host plants. In the present study, the widely cultivated watermelon cultivar Zaojia 84–24, which is sensitive to Fusarium wilt, and the wheat straw D125 were used to investigate the origination of disease resistance by RNA-Seq technology and physiological test. Our results provide important insights into the underlying molecular response of the wheat straw induced enhancement of watermelon resistance to Fusarium wilt. Our findings serve as a basis for further research on mechanism of plant disease resistance.

## Results

### Effects of wheat straw on the incidence, disease severity and plant growth of monoculture watermelon

**Fig. 1:**
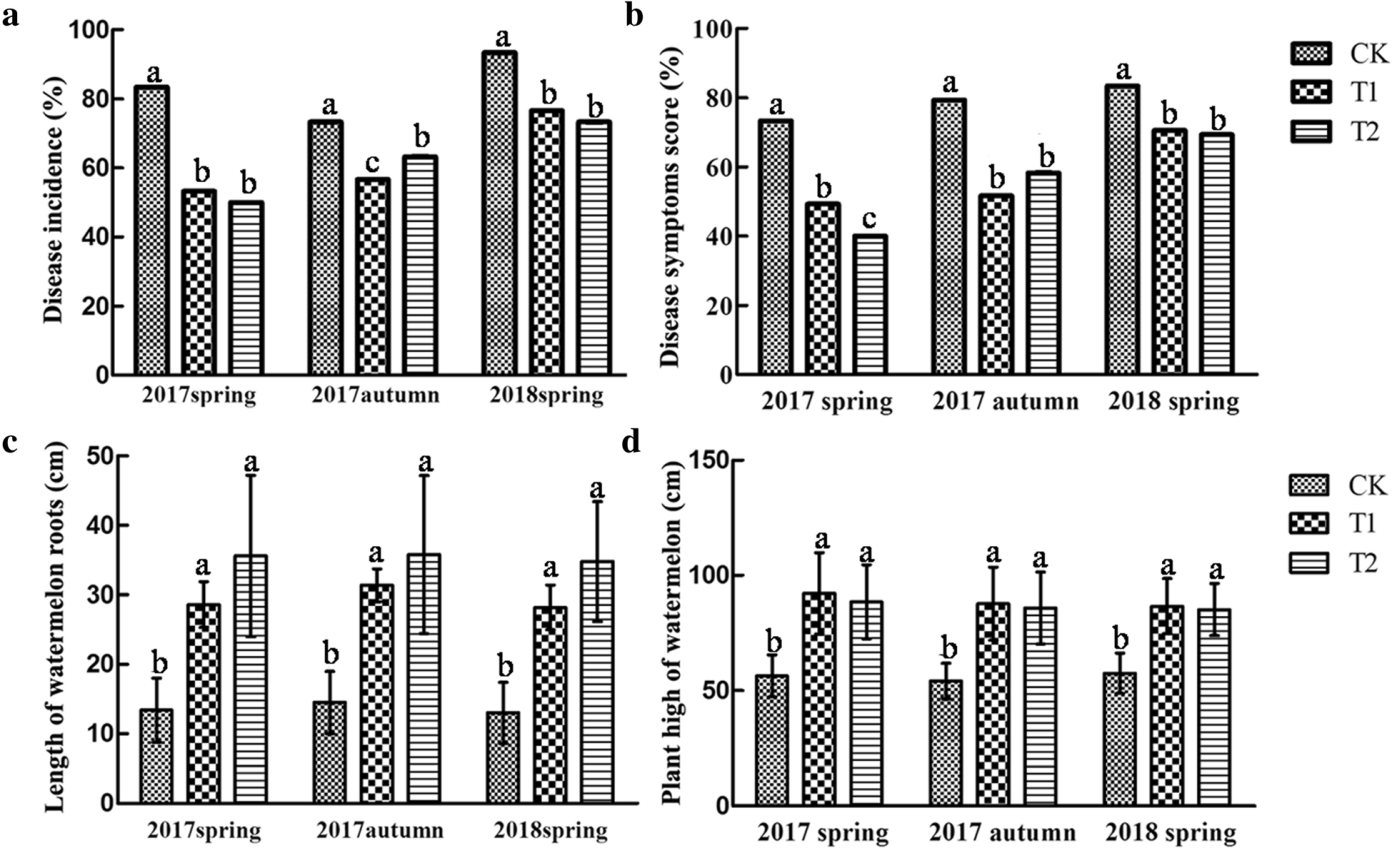
Effects of wheat straw addition on the disease incidence (**a**), disease symptoms score (**b**), root length (**c**) and plant height (**d**) of monoculture watermelon at different times points. The data are the means of 20 replicates, with standard errors shown by the vertical bars. The letters above the bars (a, b, or c) represent the different groups according to Tukey’s test at the *p* = 0.05 level. CK, without adding wheat straw; T1, addition of 1% wheat straw; T2, addition of 2% wheat straw

In the pot experiments, the effects of the three treatments (CK, without adding wheat straw; T1, adding 1% wheat straw; T2, adding 2% wheat straw) on the disease incidence and plant growth of monoculture watermelon were assessed. The disease incidences in T1 and T2 were all significantly lower than in CK at different time points, and that in T1 was significantly lower than in T2 and CK in the autumn of 2017 experiment. However, no significant differences occurred between T1 and T2 in the spring of 2017 and spring of 2018 (Fig. 1a). The trend of the disease index was similar to that of the disease incidence (Fig. 1b). In addition, the root length was longer and plant height was taller in T1 and T2 than that in CK at each time point, while the root length and plant height in T1 and T2 were similar (Fig. 1c, d). These results showed that wheat straw reduced the disease incidence of monoculture watermelon, and promoted watermelon growth, and it was no correlated with the amount of wheat straw addition.

### Effects of wheat straw addition on Fon1 population in rhizosphere soil

**Fig. 2:**
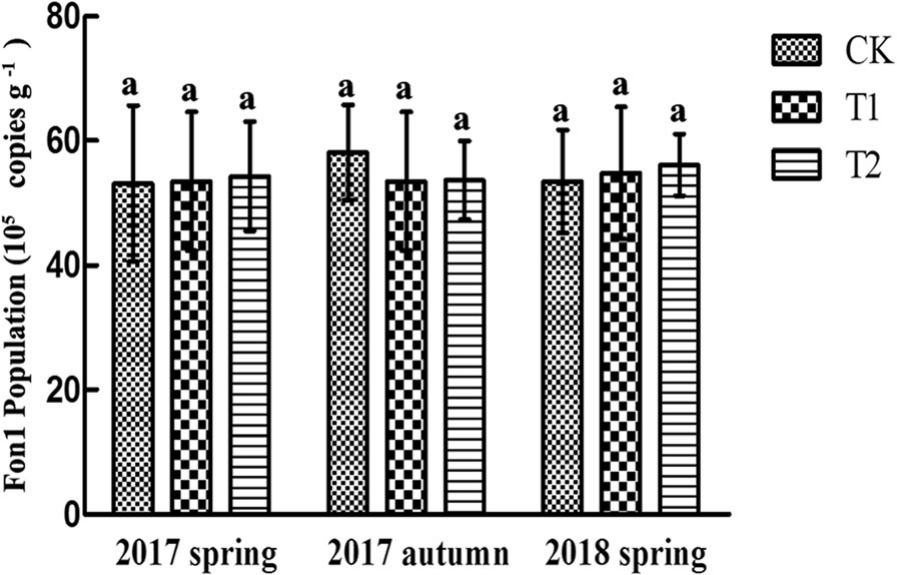
Effects of wheat straw addition on the abundance of *Fusarium oxysporum* f. sp. *niveum* in the rhizosphere of monoculture watermelon at different times points. The data are the means of three replicates, with standard errors shown by the vertical bars. The letters above the bars represent the different groups according to Tukey’s test at the *p* = 0.05 level. CK, without adding wheat straw; T1, addition of 1% wheat straw; T2, addition of 2% wheat straw

DNA was collected from the watermelon rhizosphere soil of different treatments and the copy number of *Fon1* was determined by real-time PCR. No significant difference was observed in Fon1 population among the three treatments (Fig. 2). Furthermore, wheat straw decomposing had no effect on Fon1 mycelia growth and spore germination (Additional file 1: Figure S1A and B, Table S1). These results indicated that wheat straw had no inhibitory effect to pathogens in monoculture watermelon soil and that the wheat straw decomposing had no effect on Fon1 growth and germination.

### RNA-Seq analysis and alignment to the reference genome

As shown in Fig. 1a, the biggest difference of disease incidence between the CK group and the T1/T2 group was observed in the spring of 2017 experiment. Therefore, the watermelon roots in spring of 2017 were selected for RNA-Seq analysis.

Nine RNA-Seq libraries, including three in CK group (CK-1, CK-2 and CK-3), three in T1 group (T1–1, T1–2 and T1–3), and three in T2 group (T2–1, T2–2 and T2–3), were sequenced. After quality control and data filtering of the raw sequences, a total of 495 million clean paired-end reads (≥ 150 bp) were selected for further analysis. These clean reads were deposited in the Sequence Read Archive Database (Accession SRP158956) of the National Center for Biotechnology Information (NCBI). The reads of all samples were mapped to the high-quality watermelon reference genome (Additional file 2: Figure S2). The mapping percentage is 98.55% and a standard deviation is 1.1% (Table 1).

**Table 1:** Number of clean reads that were generated from each sample and that were sequenced and mapped to the 97,103 genome

Sample name	Total No. clean reads	Reads mapped	Percentage of mapped reads (%)
CK-1	56,270,632	56,270,632	98.65%
CK-2	51,565,100	51,565,100	98.65%
CK-3	47,408,908	47,408,908	98.63%
T1–1	48,991,374	48,991,374	98.61%
T1–2	58,394,970	58,394,970	98.51%
T1–3	46,693,738	46,693,738	98.57%
T2–1	62,248,678	62,248,678	98.57%
T2–2	62,655,134	62,655,134	98.33%
T2–3	60,669,156	60,669,156	98.41%

### Verification of the RNA-Seq results

**Fig. 3:**
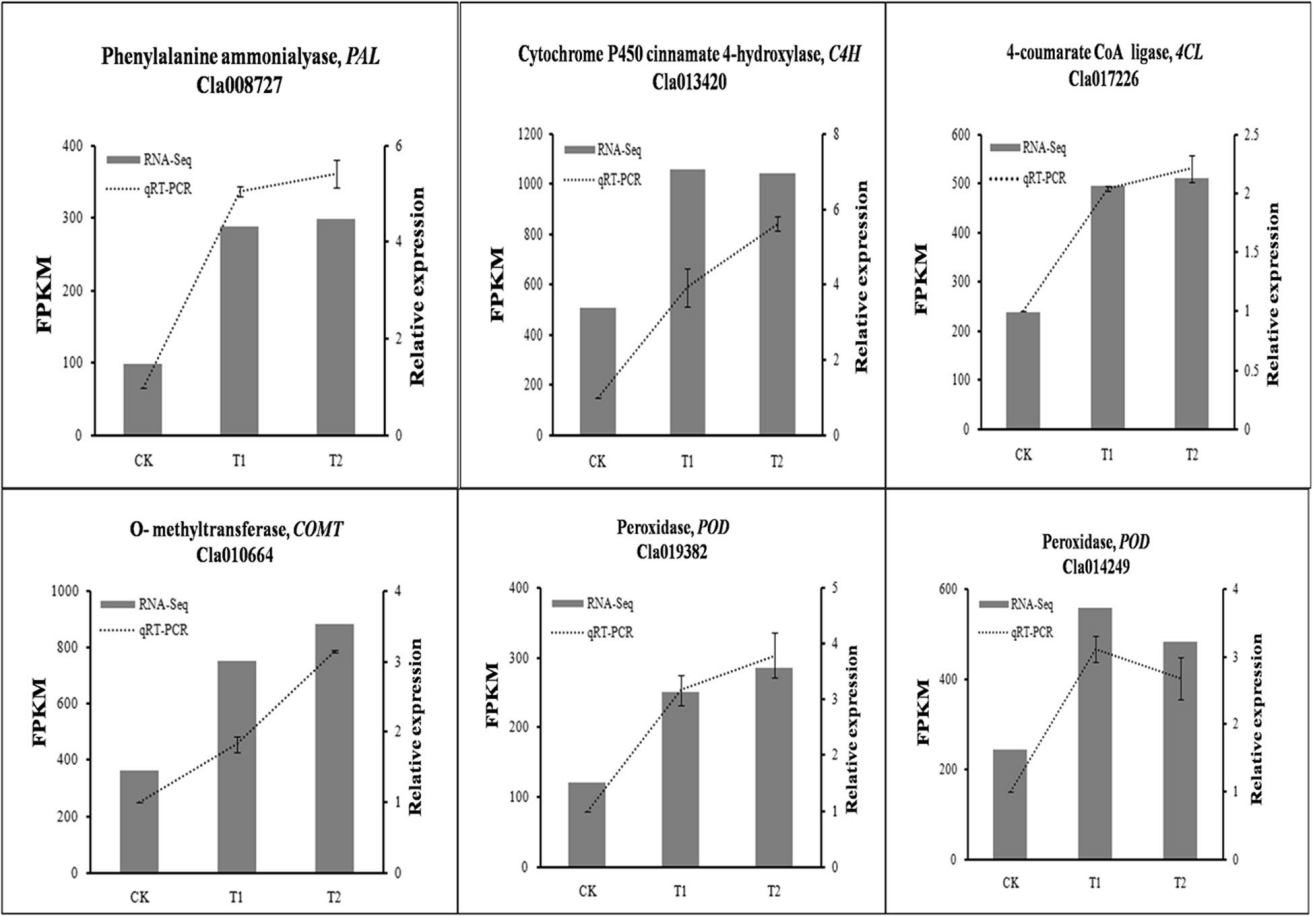
Effects of wheat straw addition on the expression levels of lignin biosynthesis in watermelon roots. Y-axis (left) indicates RNA-Seq data; Y-axis (right) indicates qRT-PCR data. Data from qRT-PCR are means of three replicates and bars represent SE. Data from RNA-seq are means of the replicates. CK, without adding wheat straw; T1, addition of 1% wheat straw; T2, addition of 2% wheat straw

**Fig. 4:**
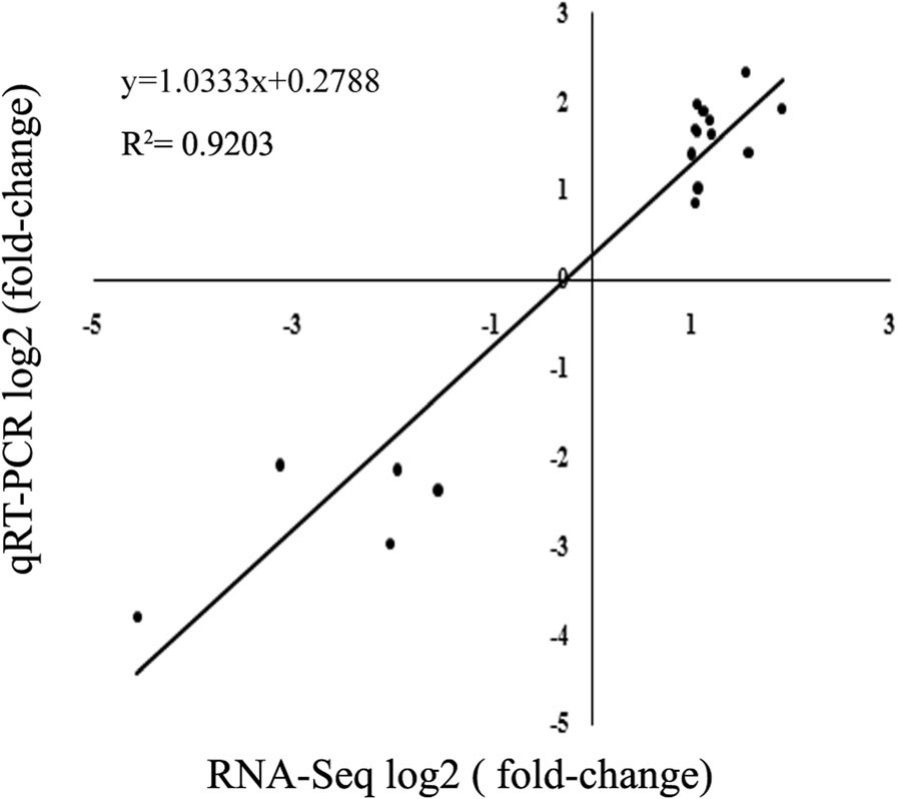
Correlation of expression levels between RNA-Seq and qRT-PCR in watermelon roots

Quantitative real-time PCR (qRT-PCR) was used to verify the reliability of the transcriptome analysis, using 17 gene-specific primers, including 11 phenylpropanoid metabolite pathway genes, 1 SAUR gene and 5 other downregulated genes (Additional file 3: Table S2). Our results showed that the expression patterns of PAL (*Cla008727*), C4H (*Cla013420*), 4CL (*Cla017226*), COMT (*Cla010664*), POD (*Cla016012* and *Cla014249*) were consistent with the sequencing results (Fig. 3). A significant strong positive correlation between the qRT-PCR and RNA-Seq data was observed (*R*^*2*^ = 0.9203) (Fig. 4, Additional file 4: Figure S3).

### Analysis of differentially expressed genes (DEGs) in the roots of monoculture watermelon in response to wheat straw

**Fig. 5:**
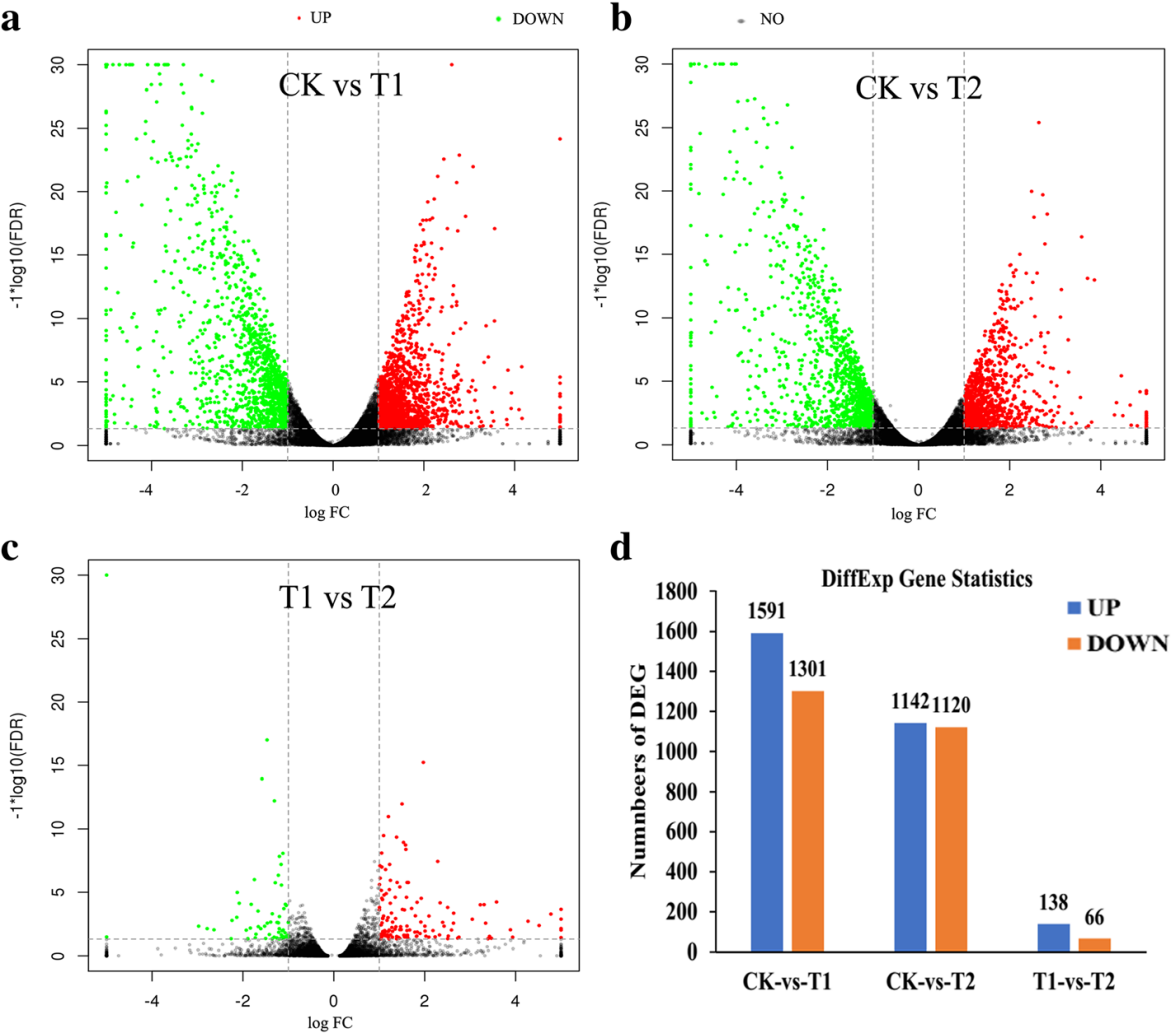
Gene expression levels in volcano plots (**a**, **b** and **c**) and different gene expression statistics (**d**). The X-axis indicates the logarithm of the difference between the two subgroups (**a**, **b** and **c**), and the Y-axis indicates the negative log10 value of the FDR for the two subgroups (**a**, **b** and **c**). The red dots represent upregulated expression, the green dots represent downregulated expression and the black dots represent no difference (**a**, **b** and **c**). CK, without adding wheat straw; T1, addition of 1% wheat straw; T2, addition of 2% wheat straw

For all treatments, a stringent valve of FDR < 0.05 and absolute value of |log_2_FC| > 1 were used as threshold for categorizing the DEGs and identifying significant differences in gene expression between CK and T1, CK and T2, T1 and T2 (Additional file 5: Table S3). Figure 5a and d showed that there were 2892 DEGs between CK and T1, among which 1591 were upregulated and 1301 were downregulated in terms of their expression. Two thousand two hundred sixty-two DEGs between CK and T2 were identified, including 1142 upregulated and 1120 downregulated (Fig. 5b and d). Only 204 DEGs between T1 and T2 were identified, among which 138 were upregulated and 66 were downregulated (Fig. 5c and d). These results suggested the addition of 1% or 2% wheat straw has little effect on the relative gene expression patterns in the watermelon roots.

### GO analysis of the DEGs

**Fig. 6:**
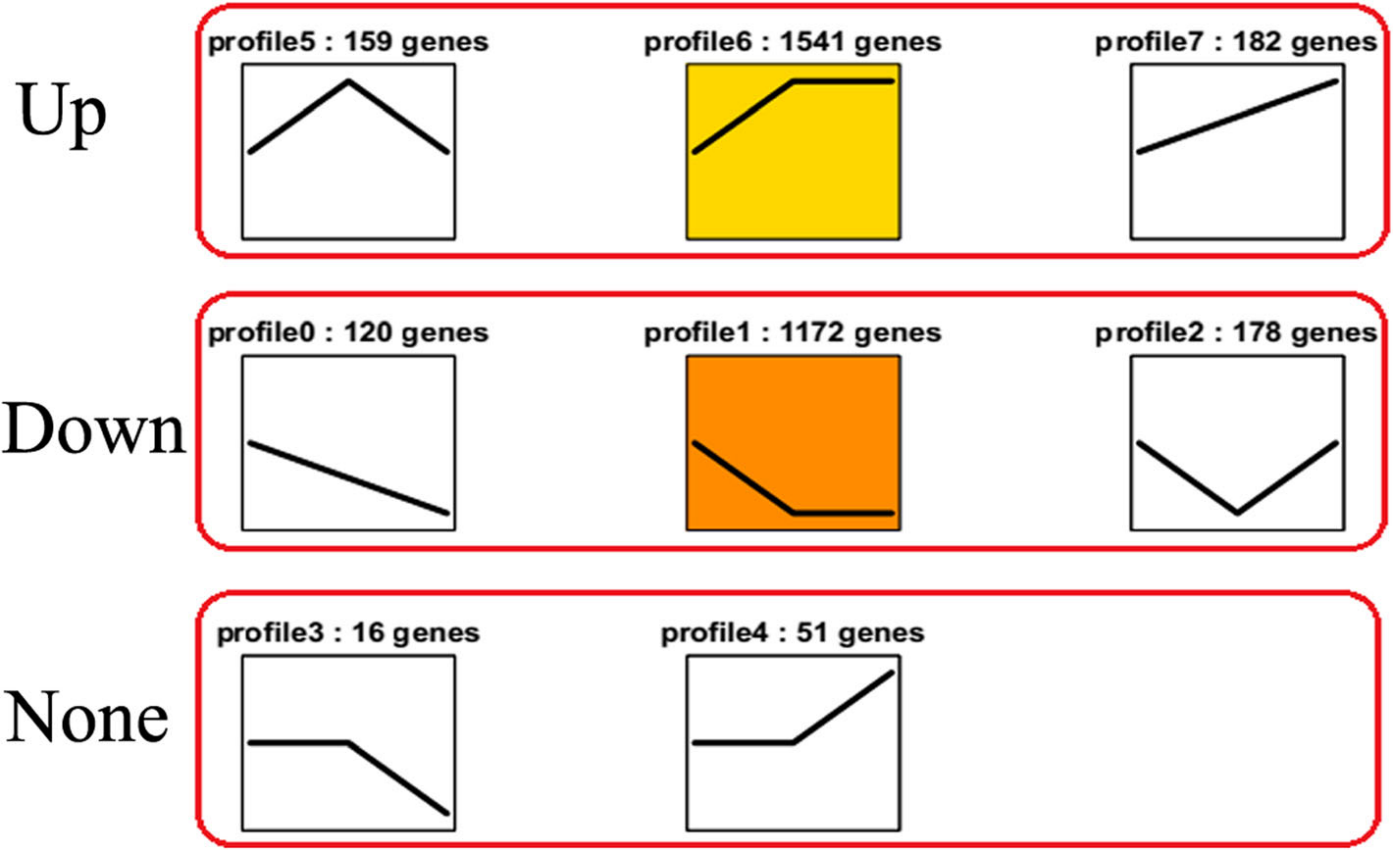
DEGs expression profiles. “Up” represents upregulated expression trends (profile 5, profile 6 and profile 7), “Down” represents downregulated expression trends (profile 0, profile 1 and profile 2) and “None” represents no change in expression trends (profile 3 and profile 4). The profile number follows the number of DEGs (top)

**Fig. 7:**
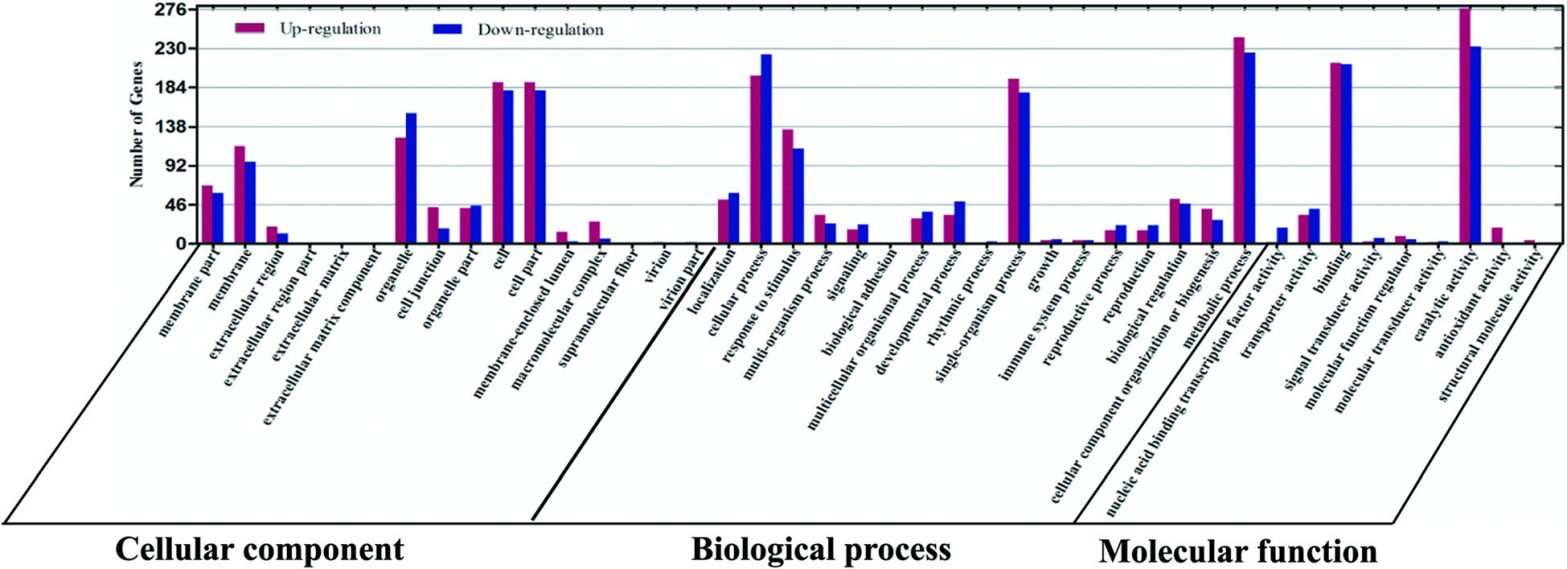
GO functional classification of DEGs. The X- axis represents the functional items, and the Y- axis represents the number of genes

Using Short Time-Series Expression Miner (STEM), a total of 3419 DEGs were clustered into 8 profiles [22]. The eight profiles (*p* < 0.05) included three groups. The first group included three types of early upregulated patterns: profile 5, profile 6 and profile 7. The second group included three types of early downregulated patterns: profile 0, profile 1 and profile 2. The last group included two types of early expression genes whose patterns did not change: profile 3 and profile 4 (Fig. 6). Specifically, 2714 DEGs were clustered into two profiles, which included one downregulated pattern (profile 1) and one upregulated pattern (profile 6). Because profile 6 and profile 1 included more DEGs than the other profiles, the DEGs were significantly enriched in profile 6 and profile 1. The DEGs within profile 1 and profile 6 were further analyzed using GO term analysis. These DEGs were allocated to three core categories: cellular component, biological process, and molecular function. Of the cellular component group, cell, cell part, organelle, membrane and membrane part were the most abundant GO terms. Of the biological process group, majority of the DEGs were classified into metabolic process, cellular process, response to stimulus and single-organism process. Under the molecular function group, catalytic activity and binding were the most abundant GO terms, followed by transporter activity, nucleic acid binding transcription factor activity and antioxidant activity (Fig. 7).

### KEGG pathway analysis of DEGs

**Fig. 8:**
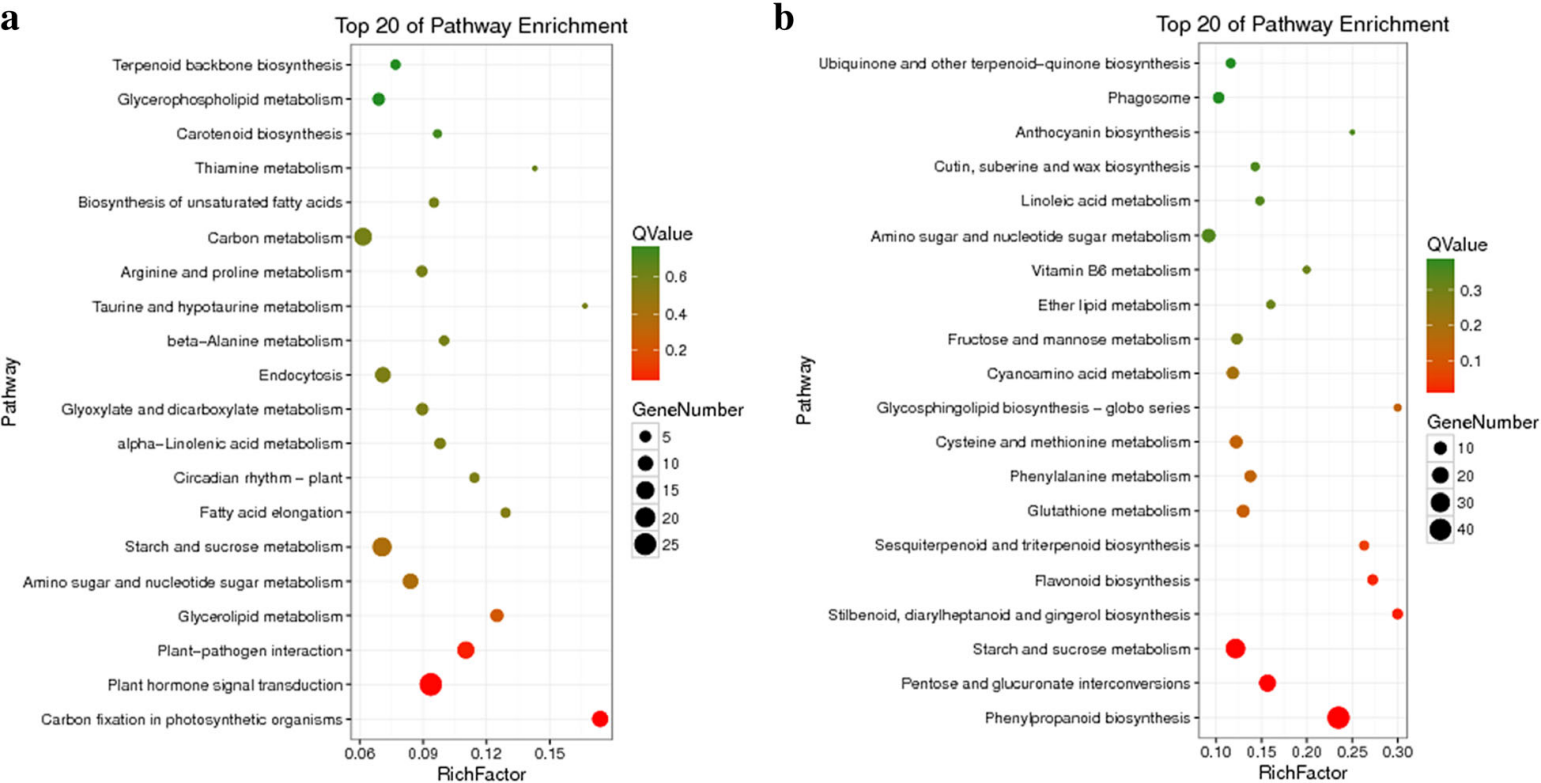
Bubble plot of the KEGG pathway enrichment of DEGs. The top 20 of enriched pathways for profile 1 with downregulated patterns (Q value < 0.05) (**a**) and profile 6 with upregulated patterns (Q value < 0.05) (**b**). Bubble color and size correspond to the Q value and gene number enriched in the pathway. The rich factor indicates the ratio of the number of DEGs mapped to a certain pathway to the total number of genes mapped to this pathway

When mapped to the reference annotation pathway in KEGG, 16.99% (581/3419) of the DEGs were annotated to 108 different metabolic pathways (Additional file 6: Table S4). The top 10 highly enriched KEGG pathways were annotated and they were present in all 8 profiles (Table 2). Of these KEGG pathways, the starch and sucrose metabolism (ko00500) were the most abundant term, constituting 10.50% (61/581) of the DEGs. The other highly enriched pathways included phenylpropanoid biosynthesis (ko00940), plant hormone signal transduction (ko04075), pentose and glucuronate interconversions (ko00040), amino sugar and nucleotide sugar metabolism (ko00520), protein processing in endoplasmic reticulum (ko04141), plant-pathogen interaction (ko04626), carbon metabolism (ko00900), biosynthesis of amino acids (ko01230) and endocytosis (ko04144). For profile 6, the significantly enriched DEGs were in the first 20 pathways (Fig. 8b, Additional file 7: Table S5b). The most enriched pathway with the largest number of DEGs was phenylpropanoid biosynthesis pathway. Phenylpropanoid biosynthesis was also among the most notable enriched pathways with the lowest Q value. Similar to profile 6, the DEGs were significantly enriched in the first 20 pathways in profile 1 (Fig. 8a, Additional file 7: Table S5a). Plant hormone signal transduction pathway had both the largest number of DEGs as well as the lowest Q value. These results showed phenylpropanoid biosynthesis and plant hormone signal transduction are the key metabolic pathways in wheat straw in response to Fusarium wilt.

**Table 2:** Top 10 KEGG pathways in termS of representation of DEGs

Pathways	NO. DEGs with pathway annotation									Pathway ID
	All profile (% of 581)	Profile 0 (% of 11)	Profile 1 (% of 192)	Profile 2 (% of 27)	Profile 3 (% of 6)	Profile 4 (% of 21)	Profile 5 (% of 31)	Profile 6 (% of 248)	Profile 7 (% of 45)	
Starch and sucrose metabolism	61 (10.50%)	1 (9.09%)	18 (9.38%)	2 (7.41%)	1 (16.67%)	3 (14.29%)	2 (6.45%)	31 (12.50%)	3 (6.67%)	ko00500
Phenylpropanoid biosynthesis	57 (9.81%)	3 (27.27%)	3 (1.56%)	1 (3.70%)	1 (16.67%)	0 (0.00%)	3 (9.68%)	43 (17.34%)	3 (6.67%)	ko00940
Plant hormone signal transduction	48 (8.26%)	2 (18.18%)	26 (13.54%)	1 (3.70%)	0 (0.00%)	1 (4.76%)	2 (6.45%)	15 (6.05%)	1 (2.22%)	ko04075
Pentose and glucuronate interconversions	31 (5.34%)	0 (0.00%)	6 (3.12%)	0 (0.00%)	0 (0.00%)	0 (0.00%)	2 (6.45%)	21 (8.47%	2 (4.44%)	ko00040
Amino sugar and nucleotide sugar metabolism	31 (5.34%)	1 (9.09%)	11 (5.73%)	3 (11.11%)	0 (0.00%)	2 (9.52%)	2 (6.45%)	12 (4.84%)	0 (0.00%)	ko00520
Protein processing in endoplasmic reticulum	28 (4.82%)	0 (0.00%)	4 (2.08%)	0 (0.00%)	0 (0.00%)	5 (23.81%)	0 (0.00%)	6 (2.42%)	13 (28.89%)	ko04141
Plant-pathogen interaction	27 (4.65%)	1 (9.09%)	15 (7.81%)	0 (0.00%)	1 (16.67%)	1 (4.76%)	1 (3.23%)	9 (3.63%)	1 (2.22%)	ko04626
Carbon metabolism	23 (3.96%)	0 (0.00%)	5 (2.60%)	2 (7.41%)	1 (16.67%)	0 (0.00%)	0 (0.00%)	16 (6.45%)	0 (0.00%)	ko00900
Biosynthesis of amino acids	22 (3.79%)	0 (0.00%)	11 (5.73%)	0 (0.00%)	0 (0.00%)	1 (4.76%)	1 (3.23%)	6 (2.42%)	0 (0.00%)	ko01230
Endocytosis	17 (2.93%)	0 (0.00%)	5 (2.60%)	0 (0.00%)	2 (33.33%)	1 (4.76%)	1 (3.23%)	10 (4.03%)	1 (2.22%)	ko04144

### Wheat straw activates the phenylpropanoid metabolic pathway and enhances lignin synthesis

**Fig. 9:**
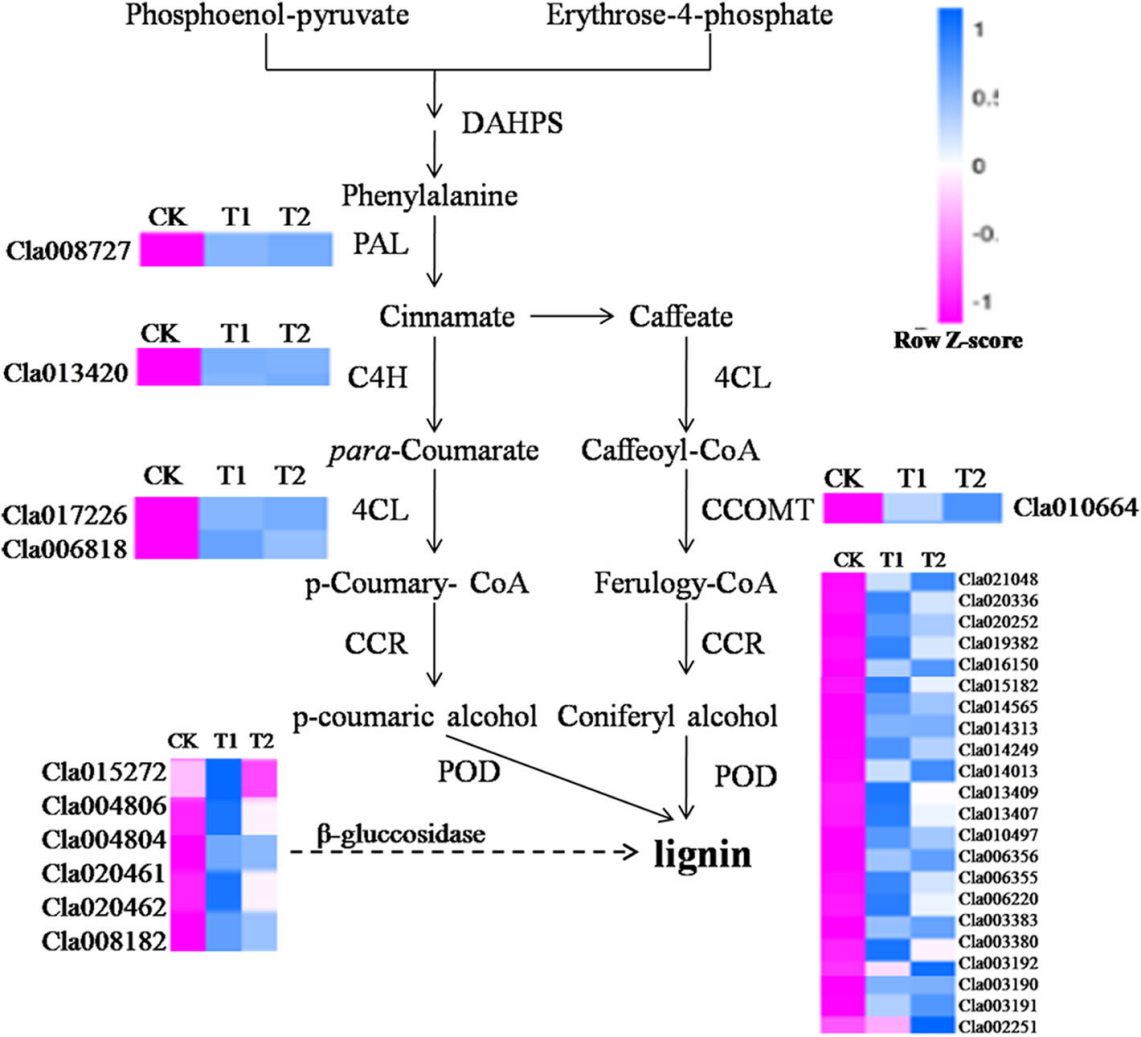
The partial lignin biosynthesis pathway that differentially expressed genes involved. CK, without adding wheat straw; T1, addition of 1% wheat straw; T2, addition of 2% wheat straw

The phenylpropanoid metabolic pathway is an early indicator for plant defense. RNA-Seq analysis showed a total of 57 DEGs were associated with the phenylpropanoid biosynthesis pathway. Fourteen of those encoded phenylalanine ammonia-lyase (*PAL*, *Cla008727*), trans-cinnamate-4-monooxygenase (*C4H*, *Cla013420*), shikimate O-hydroxycinna-moyl transferase (*HCT*, *Cla022713* and *Cla009995*), 4-coumarate-CoA ligase (*4CL*, *Cla017226* and *Cla006818*), caffeoylshikimate esterase (*CSE*, *Cla012691* and *Cla006446*), coumaroylquinate 3′-monooxygenase (*C3H*, *Cla017432*), caffeic acid 3-O-methyltransferase (*COMT*, *Cla010664*), caffeoyl-CoA-O-methyltransferase (*CCoAOMT*, *Cla016012*), ferulate-5-hydroxylase (*F5H*, *Cla014265*), and coniferyl-aldehyde dehydrogenase (*REF1*, *Cla012342*); 22 encoded peroxidase (POD) and 7 encoded beta-glucosidas, all of which were clustered into profile 6 (Table 3, Additional file 8: Figure S5A and B). Furthermore, expression of PAL, C4H, 4CL, COMT and POD was up-regulated in T1 and T2 compared with CK. These enzymes are all involved in the biosynthesis of lignin [23]. Their expression levels were peaked in T1 and maintained at high level in T2 (Fig. 9). The expression level of beta-glucosidas, which plays an important role in cellulose degradation [24] was also significantly upregulated in T1 compared with T2 and CK, and the expression was higher in T2 than CK.

**Table 3:** Expression patterns of DEGs related to lignin biosynthesis

Component	All profiles	Profile 6	Profile1	Description
Phenylpropanoid biosynthesis	57	43	3	
*PAL*	1	1	0	Phenylalanine ammonia-lyase
*C4H*	1	1	0	Trans-cinnamate 4-monooxygenase
*4CL*	2	2	0	4-Coumarate-CoA ligase
*HCT*	2	2	0	Shikimate O-hydroxycinnamoyl transferase
*CSE*	2	2	0	Caffeoylshikimate esterase
*C3H*	1	1	0	Coumaroylquinate 3′-monooxygenase
*COMT*	1	1	0	Caffeic acid 3-O-methyltransferase
*CCoAMT*	1	1	0	Caffeoyl-CoA-O-methyltransferase
*F5H*	2	1	1	Ferulate-5-hydroxylase
*REF1*	1	1	0	Coniferyl-aldehyde dehydrogenase
*Beta-glucosidase*	11	7	1	*Beta-glucosidase*
*Peroxidase*	30	22	1	*Peroxidase*

**Fig. 10:**
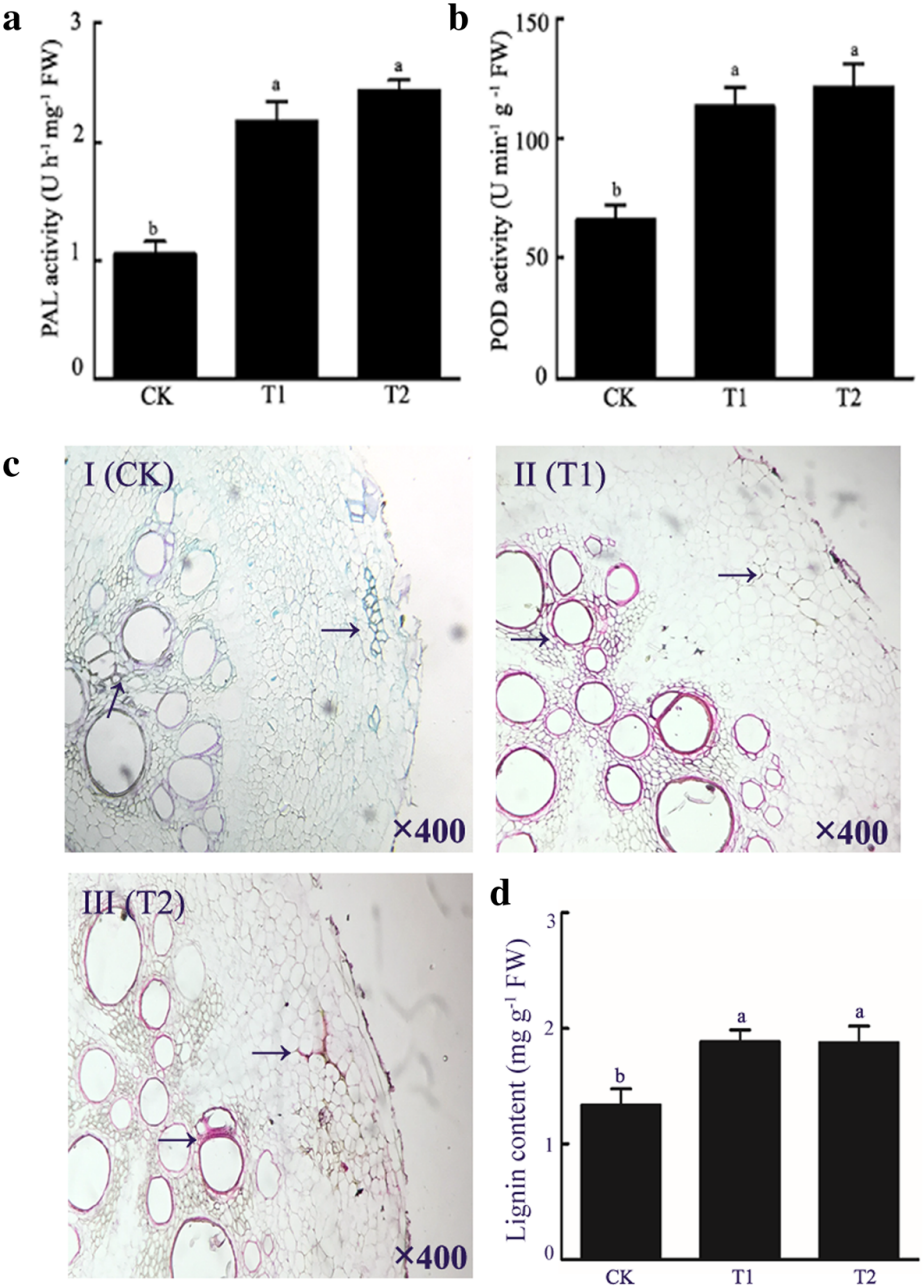
Effects of wheat straw addition on the content and distribution of lignin in watermelon roots. PAL activity (**a**) and POD activity (**b**) in watermelon roots. Cross-sections of the main roots of watermelon at flowering stage, stained with Safranin O (red) and Fast green FCF (green) (**c**). Arrows indicate the lignin distribution in watermelon roots. Magnification = 4400×. The lignin conent in watermelon roots (**d**). CK, without adding wheat straw; T1, addition of 1% wheat straw; T2, addition of 2% wheat straw

The activities of PAL and POD were further measured and the results showed that PAL and POD activities were increased in T1 and T2 compared with CK, and there no difference between T1 and T2 (Fig. 10a and b). To further verify this observation, the distribution of lignin in the root tips of watermelon in each treatment was examined by light microscopy (Fig. 10c). More lignin was observed in cell walls of both cortical and vascular bundle cells of T1 (Fig. 10cII) and T2 (Fig. 10cIII) than in CK (Fig. 10cI). Quantification of lignin content further showed that the lignin content was significantly higher in T1 and T2 than in CK, while no difference between T1 and T2 (Fig. 10d). The above results showed that the wheat straw activated the phenylpropanoid metabolic pathway and induced the lignin biosynthesis.

### Wheat straw activates auxin biosynthesis

KEGG analysis revealed that 48 DEGs were involved in signal transduction pathways of plant hormones, including auxin, cytokinine, gibberellins, abscisic acid (ABA), ethylene (ET), brassinosteroid (BRs), jasmonic acid (JA) and salicylic acid (SA) (Table 4, Additional file 9: Figure S6).

**Table 4:** List of important DEGs involved in plant hormone signal transduction

Components	All profiles	Profile 1	Profile 6
Auxin			
AUX1	1	0	1
AUXIAA	6	5	1
SAUR	6	0	4
Cytokinin			
B-ARR	1	0	1
A-ARR	2	1	1
Gibberellins			
TF	1	1	0
DELLA	1	0	1
Abscisic acid			
PYR/PYL	5	1	4
PP2C	1	1	0
ABF	1	2	0
SnRK	2	0	1
Ethylene			
E-BF1/2	4	1	2
Brassinosteroids			
BSK	2	1	1
TCH4	1	1	0
BAK1	1	0	1
CYCD3	1	0	1
Jasmonic acid			
JAR1	1	1	0
JAZ	5	1	0
MYCZ	1	1	0
Salicylic acid			
TGA	1	1	0
PR-1	1	0	0

**Fig. 11:**
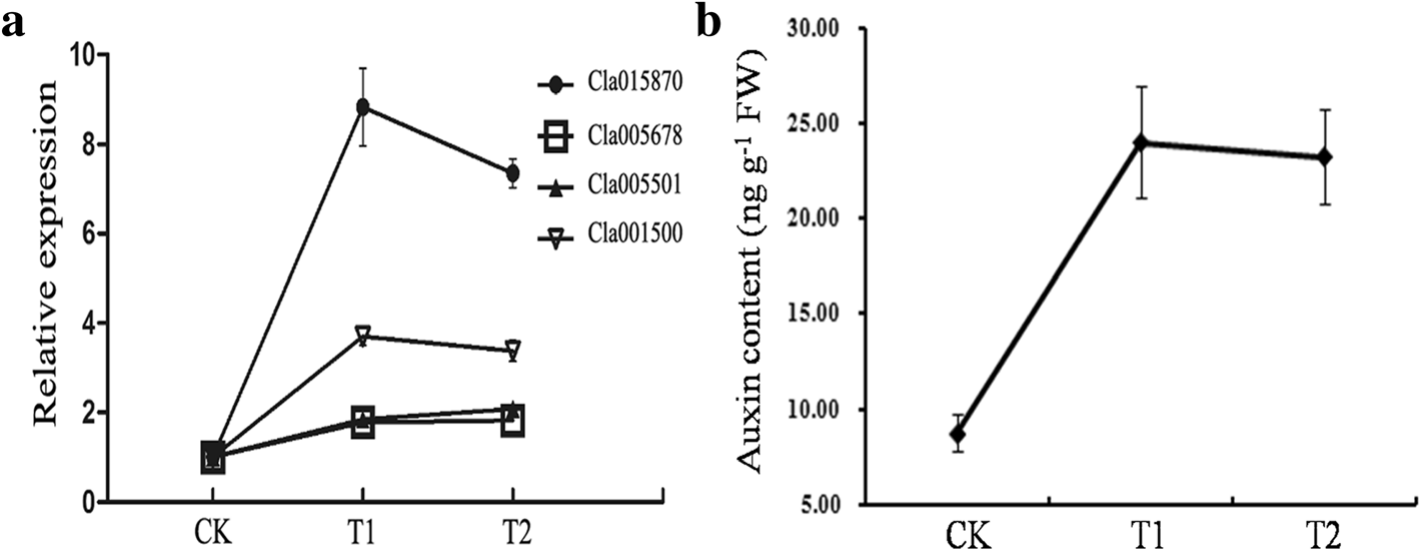
Effects of wheat straw addition on the expression levels of SAUR (**a**) and the content of auxin (**b**) in watermelon roots. CK, without adding wheat straw; T1, addition of 1% wheat straw; T2, addition of 2% wheat straw

In the signal transduction pathway for auxin, 6 out of the 13 DEGs, including an auxin influx carrier (*AUX1*, *Cla006581*), an auxin responsive protein (*AUXIAA*, *Cla007545*) and four small auxin-up RNA (SAUR) family proteins (*SAUR*, *Cla015870*, *Cla005678*, *Cla005501* and *Cla001500*), were clustered into profile 6 and were upregulated. Five *AUXIAA* (*Cla021419*, *Cla014808*, *Cla012649*, *Cla007544* and *Cla002975*) were clustered to profile 1 and showed downregulated trend. In addition, the SAUR may be the largest family of early auxin-response genes and is widespread among land plant species [25]. Cla015780 expression was 8.8 and 7.8 times higher in T1 and T2 than in CK, respectively (Fig. 11a). The auxin content was also higher in T1 and T2 than in CK (Fig. 11b). Similar results were observed in the ET, ABA, cytokinine and BR signal transduction pathways. In JA transduction pathway, 3 out of the 7 DEGs showed downregulated trends (profile 1), while no DEGs showed upregulated tends (profile 6). In SA signal pathway, only 1 DEG that encode a transcription factor TGA (*TGA*, *Cla007982*) showed a downregulated trend while no DEGs showed upregulated trends. In addition, the DEGs for phytochrome-interacting factor 4 (*PTF4*, *Cla023247*) was down-regulated for DELLA protein (*DELLA*, *Cla012302*) was upregulated. In gibberelline signal pathway, no difference was observed between the upregulated and downregulated DEGs. The above results showed that wheat straw activated the auxin biosynthesis, but showed little effect on disease resistance genes of JA and SA metabolic pathways. We speculate that wheat straw dose not induce resistance of monoculture watermelon, but enhances the base defense response by promoting the growth of monoculture watermelon.

## Discussion

Physiological and biochemical studies have shown that most of the *Fusarium* resistant watermelon varieties have strong cell structure, such as thickened xylem to prevent entry of the pathogen [26]. Lignin is one of the essential constituents of the secondary cell wall, which forms a physical barrier that increase the resilience to pathogen infection [27–29]. In our present study, analysis of root lignin content between the treatment and control groups showed that the lignin content in roots of the T1 and T2 groups was insignificantly higher compared to the roots of control group (Fig. 10d). The results indicated that wheat straw may restrict Fon1 infection to watermelon roots. Moreover, histochemical analyses showed that the lignin distribution of watermelon root was significantly increased in treatment groups compared to the control group (Fig. 10c), further suggesting that lignin enrichment could enhance the cell wall structure and the resistance to Fon1 infection.

Secondary metabolites, such as lignin, play a crucial role in defending pathogen infection in plants. Lignin biosynthesis is a complex process that requires the participation of many enzymes [30]. PAL is one of the key enzymes and also the first rate-limiting enzyme in the phenylpropanoid pathway [31]. Kim et al. [32] reported that PAL is closely related to plant defense and is induced by pathogens. POD is a heme-containing enzyme that catalyzes many different substrates using H_2_O_2_ as an oxidant [33]. Overexpression of a tomato basic peroxidase significantly increases the lignin content [34, 35]. In our study, RNA-Seq analysis showed that several lignin biosynthesis related genes were upregulated in the treatment groups (T1 and T2). DEGs analysis revealed that the expression of genes involved in phenylpropanoid metabolic pathway was also increased in the treatment groups compared with the control group (Additional file 8: Figure S5A). These observations were confirmed by qRT-PCR analysis of *PAL*, *POD*, *4CL*, *C4H* and *CCOMT* genes (Fig. 3), which are the key genes in lignin biosynthesis. Inhibition of *4CL* expression or *PAL* or *C4H* expression significantly reduced the lignin content in transgenic tobacco [36, 37].

Plant hormones are important factors in regulating both plant growth and responses to various stresses. Auxin is believed to regulate or influence stress responses at the whole-plant level [38, 39], and its biosynthesis and signaling are closely linked to SAUR proteins [40]. For example, the SAUR gene *Bra019369* had a higher expression in pathogen resistant plant compared to pathogen sensitive plant in canola [41]. *OsSAUR45* was also involved in rice growth through affecting auxin synthesis [42]. In the current study, *Cla015870*, which encodes a SAUR family protein, was highly induced in T1 relative to CK (10.4 folds) and T2 relative to CK (9.8 folds) (Additional file 5: Table S3), respectively. This result suggests that the wheat straw promoted watermelon growth may be related to the upregulation of auxin synthesis genes.

JA, SA and ET play a vital role in host resistance against biotrophic pathogens [43–48]. In the current study, however, GO terms related to JA and ET biosynthesis, metabolism or signaling processes were not significantly changed and the SA biosynthesis was downregulated in T1 and T2 compared to in CK (Table 4). It is possible that wheat straw did not induce SA, JA and ET signaling pathways in response to Fon1 infection in monoculture watermelon.

To further confirm whether lignin can hinder Fon1 infesting the watermelon roots, the absolute expression of *Fon1* was measured in watermelon roots. Results showed that the absolute expression of *Fon1* was lower in T1 and T2 than in CK (data not shown). It is clear that the addition of wheat straw prevents the entry of Fon1 into the watermelon roots. The above results suggest that the addition of wheat straw could increase lignin and auxin biosynthesis and enhance the resistance of monoculture watermelon against Fusarium wilt. However, the mechanism of wheat straw induced increase of lignin and auxin content in host plant is unclear and deserves further investigation.

## Conclusion

In conclusion, our study indicates that wheat straw could significantly enhance disease resistance against Fon1 in watermelon monoculture. Our data suggest that the addition of wheat straw triggers upregulation of the phenylpropanoid pathway-related genes, which increase the biosynthesis of lignin and auxin levels. The increased lignin and auxin enhance the cell structure, such as with thickened xylem to prevent the entry of the pathogen.

## Methods

### Experimental materials

The D125 wheat straw was kindly provided by the Vegetable Physiological Ecology Laboratory of the College of Horticulture and Landscape Architecture at Northeast Agricultural University, China. The wheat straw was naturally air dried and crushed to pieces with approximate lengths of 0.5–1 cm. Seeds of the watermelon (*Citrullus lanatus* (Thunb.) Matsum. & Nakai var. lanatus) cultivar Zaojia 84–24, were provided by the Xinjiang Academy of Agricultural Sciences, China. This watermelon cultivar is susceptible to Fusarium wilt. Monoculture watermelon soil was collected from the surface layer at the Xiangfang Farm of Northeastern Agriculture University. This farm has been used for watermelon growth for 5 years and was infected with *Fusarium oxysporum* f. sp. *niveum* race 1 (Fon1).

### Pot experiments

All pot experiments were conducted in a greenhouse at Northeast Agricultural University. Watermelon seeds were soaked with sterile distilled water for 30 min at 55 °C, then rinsed three time with water. The seeds were germinated in a mixture of peat: perlite (1:1 v/v) to their four leave stage (about 30 days). The seedlings were then transferred into plastic pots (25 cm × 25 cm) with 5 kg monoculture watermelon soil containing 0 g (CK), 50 g (T1) or 100 g (T2) of wheat straw. Each pot contained one watermelon seedling. Seven days after transplanting, the seedlings of CK, T1 and T2 groups were inoculated with 20 mL spore suspension (5 × 10^6^ spores/mL) solution of Fon1 by pouring into rhizosphere of each watermelon seedling. After including 12 h, randomly selected 5 watermelon roots were collected for RNA extract.

### Measurements of watermelon Fusarium wilt incidence, disease index, root length and plant height

Each treatment consisted of 20 pots that were randomly arranged. Plants were subjected to typical management and equal amounts of irrigation. The incidence of Fusarium wilt was observed and calculated at the flowering stage (20 days after transferred into posts), and the incidence was expressed as the percentage of diseased plants out of the total number of plants [49]. Subsequently, the plants were removed from the pots, after which the whole plants were submerged in water. The soil particles were removed gently to avoid damage to the roots. The roots were washed for measurement. A meter ruler was used to measure the distance from the cotyledon to the top of the watermelon. The experiments were repeated three times (Spring of 2017, Autumn of 2017 and Spring of 2018).

### Measurements of Fon1 population in soil

One g mix soil for randomly 5 watermelon rhizosphere soil for each treatment were collected and the genomic DNA was extracted using the PowerSoil R DNA Isolation Kit (MO BIO, USA) according to the manufacturer’s instructions and stored in a − 20 °C freezer .

Real-time PCR (RT-PCR) was used to analyze the Fon1 population. The *Fon1* primers were 5′-CGATTAGCGAAGACATTCACAAGACT-3′ and 5′-ACGGTCAAG AAGATGCAGGGTAAAGGT-3′ [50]. RT-PCR was performed using an Analytik PCR system (Analytik Jena AG, Germany). The total reaction volume was 20 μL, including 2 μL of cDNA, 0.5 μL of primer/each, 10 μL of 2 × Real SYBR Mixture (TIANGEN Biotech, China) and 7 μL of water. The PCR condition was: 94 °C for 5 min; 95 °C for 30 s, 54 °C for 30 s, 72 °C for 30 s with a total of 35 cycles. The final elongation at 72 °C for 3 min.

A target gene from Fon1 genomic DNA was used to create the plasmid standard for *Fon*1 quantification. The Fon1 DNA copy concentration was calculated as described previously [51]. PCR assays were conducted in triplicates.

### Generating and sequencing the RNA-Seq library

Watermelon roots were collected in the of Spring of 2017 for RNA-Seq analysis. Roots from 5 watermelon seedlings were collected and pooled together as one sample. Three samples were prepared from each treatment and named as CK-1, CK-2, CK-3, T1–1, T1–2, T1–3, T2–1, T2–2 and T2–3. Samples were frozen in liquid nitrogen immediately after collected and then transferred a − 80 °C freezer.

The extracted total RNA was treated with DNase I to remove all DNA residues using an RNAprep pure Plant Kit (TIANGEN, Beijing, China) following the manufacturer’s instruction. The quality, quantity and integrity of the total RNA were evaluated. The quality was evaluated using a spectrophotometer (NanoPhotometer, Implen, CA, USA); the quantity using a Fluorometer (Qubit 2.0, Life Technologies, CA, USA), and the integrity using a Bioanalyzer 2100 system (Agilent Technologies, CA, USA). After obtaining the high quality total RNA, mRNA was enriched either via oligo (dT) beads (for eukaryotic mRNA), or via removing the rRNA (for prokaryotic mRNA). The enriched mRNA was fragmented and random primers were used to reverse transcribe the mRAN into cDNA, which was used as templates to synthesize the second-strand cDNA. After purified, the cDNA fragments were subjected to end repair and addition of the poly (A) tail. A sequencing adaptor was ligated to each fragment and the ligated fragments were separated by agarose gel electrophoresis, amplified by PCR, and sequenced using an Illumina HiSeq™ 2500 system (Gene Denovo Biotech Co., Guangzhou, China).

### Transcript assembly and DEGs analysis

To obtain high quality clean reads, the raw sequencing reads were filtered and the following reads were removed from further analysis: 1.) reads containing adapters; 2.) reads containing more than 10% unknown nucleotides; and 3.) reads containing more than 50% low quality (Q-value ≤10) bases; 4.) reads containing rRNA sequences. The remaining high-quality reads were aligned against the reference transcriptome using Bowtie2 [52]. The gene abundances were calculated as reads per kb million (FPKM) [53]. The edgeR software (http://www.r-project.org/) was used to identify DEGs. Significant DEGs were identified as fold change (FC) ≥ 2 and false discovery rate (FDR) < 0.05.

### Analysis of gene ontology (GO) terms and Kyoto encyclopedia of genes and genomes (KEGG) enrichment

All identified unigenes from the watermelon roots were annotated to GO terms (http://www.geneontology.org) the based on the UniProt and the Pfam databases [54]. The annotated unigenes were then mapped to a plant-specific GO slim ontology (http://www.geneontology.org/) [55]. The Pathway Tools program [56] was used to predict the biochemical pathways of the watermelon unigenes that were arranged into the PathoLogic format.

### Determination of phenylalanine ammonia-lyase (PAL) and peroxidase (POD) enzymatic activities in watermelon roots

The PAL and POD activities were measured using a phenylalanine ammonia-lyase kit and a peroxidase kit (Ke Ming, Jiangsu, China), repectively, following the manufacturer’s instructions. The absorbance was measured at 290 nm for PAL and at 470 nm for POD using a microplate reader (Bio Tek Instruments, Inc., Highland Park, USA).

### Determination of lignin content and distribution in watermelon roots

Samples of watermelon roots were dried at 80 °C then pulverized and passed through a 40 mesh sieve. Two mg were used to measure the lignin content using a lignin content kit (Ke Ming, Jiangsu, China) following the manufacturer’s instructions. The absorbance was measured at 280 nm using a microplate reader (Bio Tek Instruments, Inc.)

Lignin distribution in the cell walls of watermelon roots was identified using light microscopy and paraffin-embedded thin sections [57]. Briefly, the watermelon roots were washed and the root tips were removed and immersed in fixing solution for 48 h. The root tips were then gradually dehydrated with ethanol and embedded in paraffin. Sections of 8–10 μm were sliced with a microtome. Sections were gradually rehydrated, stained with Safranin O and Fast Green FCF. The stained sections were observed and imaged using a light microscope (Guangzhou minghui technology Co., Guangzhou, China).

### Measurement of the SAUR gene expression and auxin content

PCR was used to measured the expression of 4 SAUR genes (*Cla001500*, *Cla015870*, *Cla005501* and *Cla005678*. Primers for these 4 genes and the control gene (actin) are listed in (Additional file 10: Table S6). The PCR conditions are: 95 °C for 10 s; 35 cycles of 95 °C for 10 s, 56 °C for 30 s and 72 °C for 30 s. Analysis of the final melting curve was performed by raising the temperature from 55 °C to 95 °C at a rate of 0.5 °C/5 s. The 2^-ΔΔCT^ method [58] was used to calculate the relative quantification of the *Fon1* expression, which was normalized to the actin gene [59]. The experiment was repeated three times using three biological replicates.

The auxin content was measured using a liquid chromatography-mass spectrometry (AB 5500, Beijing, China) as described previously [60].

### Verification of the RNA-Seq by quantitative real-time PCR (qRT-PCR)

qRT-PCR was used to validate the RNA-Seq results using a qTOWER 2.0 Real Time PCR system (Analytik Jena AG, Germany). Eleven genes that are important for lignin biosynthesis, one SAUR gene, and five randomly chosen down-regulated genes were selected. The primers were designed with Primer Premier (v 6.0) software [61] and listed in (Additional file 3: Table S2).

The total reaction volume as 20 μL, including 1 μL of cDNA template, 1 μL of each primer pair, 10 μL of SYBR Green Master mix (Toyobo, Osaka, Japan), and 8 μL of water. The PCR conditions were: 95 °C for 10 s; 35 cycles of 95 °C for 10 s, 56 °C for 30 s and 72 °C for 30 s. The experiment was repeated three times using three biological replicates.

### Statistical analysis

All data were expressed as mean ± standard errors. Statistical analysis was conducted used SPSS 16.0 software (SPSS Inc., USA) and Microsoft Excel. *p* < 0.05 was considered statistically significant.

## Supplementary information

**Additional file 1.** Colony diameter and number of germinated spores in response to different concentrations of decomposing wheat straw.

**Additional file 2.** Classification of raw reads.

**Additional file 3.** Primers used for qRT-PCR.

**Additional file 4.** Verification of RNA-Seq data.

**Additional file 5. **Fold-changes and *P* values for DEGs in the CK vs T1, CK vs T2 and T1 vs T2.

**Additional file 6.** KEGG pathway enrichment analysis of DEGs.

**Additional file 7.** KEGG pathway enrichment analysis of DEGs in profile 1 and profile 6.

**Additional file 8.** Heat map of the expression levels of DEGs annotated in the phenylpropanoid biosynthesis pathway and KEGG map.

**Additional file 9.** Heat map diagram of the expression levels of DEGs annotated in hormone signal transduction pathways by KEGG analysis

**Additional file 10.** PCR primers SAUR genes.

## Data Availability

The datasets supporting the results presented in this manuscript are included within the article (and its additional files). The raw reads for the 9 libraries sequenced are available in the NCBI Sequence Read Archive database (SRP158956).

## Abbreviations

4CL: 4-coumarate-CoA ligase
ABA: Abscisic acid
AUX1: Auxin influx carrier
AUXIAA: Auxin responsive protein
BRs: Brassinosteroid
C3H: Coumaroylquinate 3’-monooxygenase
C4H: Trans-cinnamate-4-monooxygenase
CCoAOMT: Caffeoyl-CoA-O-methyltransferase
CK: Without adding wheat straw
COMT: Caffeic acid 3-O-methyltransferase
CSE: Caffeoylshikimate esterase
DEGs: Differentially Expressed Genes
DELLA: DELLA protein
ET: Ethylene
F5H: Ferulate-5-hydroxylase
Fon1: *Fusarium oxysporum* f.sp*. niveum* race 1
GO: Gene Ontology
HCT: ShikimateO-hydroxycinna-moyl transferase
JA: Jasmonic acid
KEGG: Kyoto Encyclopedia of Genes and Genomes
PAL: Phenylalanine ammonia-lyase
POD: Peroxidase
PTF4: Phytochrome-interacting factor 4
qRT-PCR: quantitative real-time PCR
REF1: Coniferyl-aldehyde dehydrogenase
RNA-Seq: Illumina RNA sequencing
SA: Salicylic acid
SAUR: Small auxin-up RNA
STEM: Short Time-series Expression Miner
T1: Addition of 1% wheat straw
T2: Addition of 2% wheat straw
TGA: Transcription factor TGA
