# Supplementary material for: Wheat straw increases the defense response and resistance of watermelon monoculture to Fusarium wilt
Source: BMC Plant Biol. 2019 Dec 11;19:551. doi: 10.1186/s12870-019-2134-y (PMC6907359; doi:10.1186/s12870-019-2134-y)
Supplement: Supplementary file 1 — Additional file 1. Colony diameter and number of germinated spores in response to different concentrations of decomposing wheat straw. [file 12870_2019_2134_MOESM1_ESM.doc]

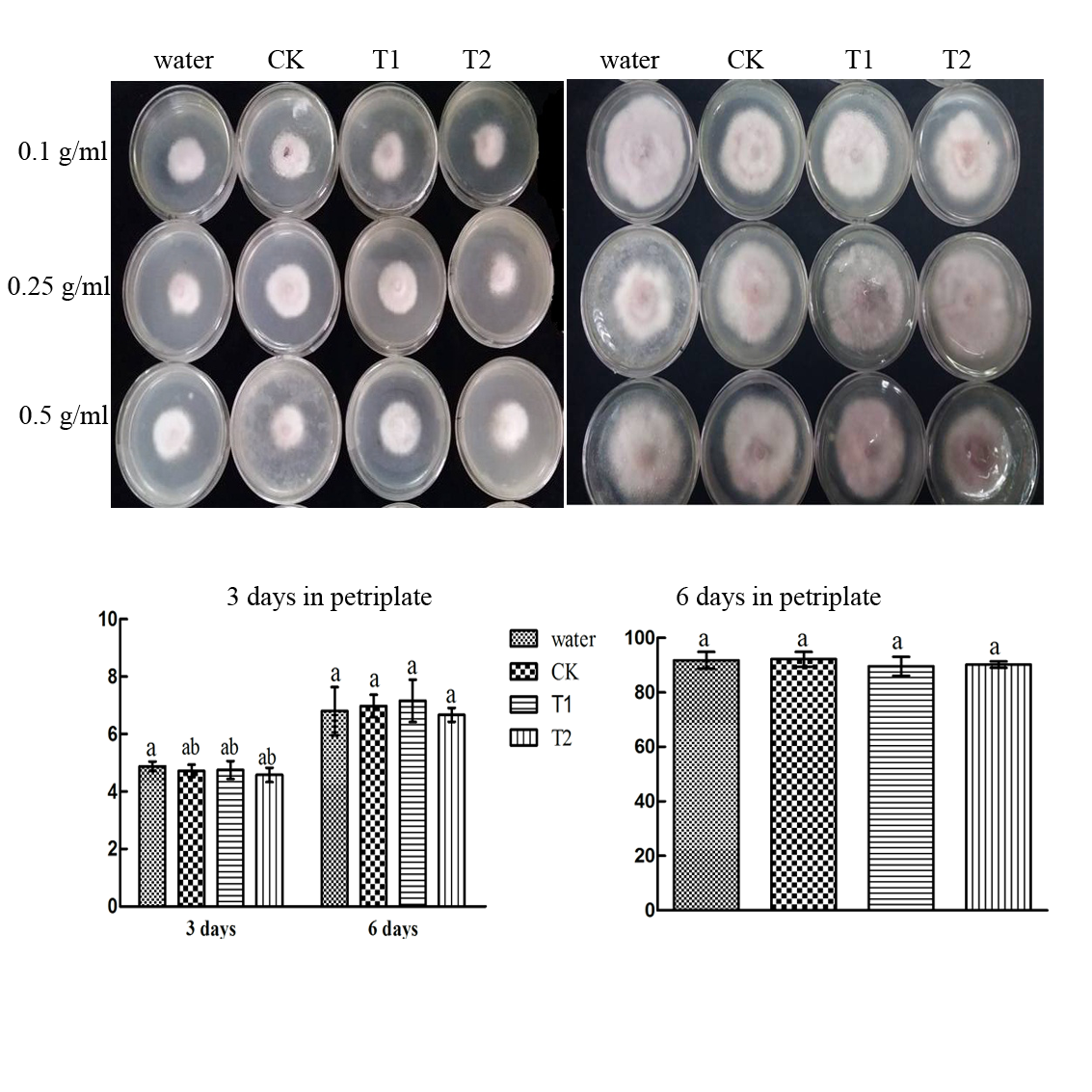


**Figure S1A** The colony diameter in different concentration of wheat straw decomposing in cultivate for 3 days and 6 days. Water, sterile water; CK, decomposing of without addition wheat straw; T1, decomposing of addition 1% wheat straw; T2, decomposing of addition 2% wheat straw.


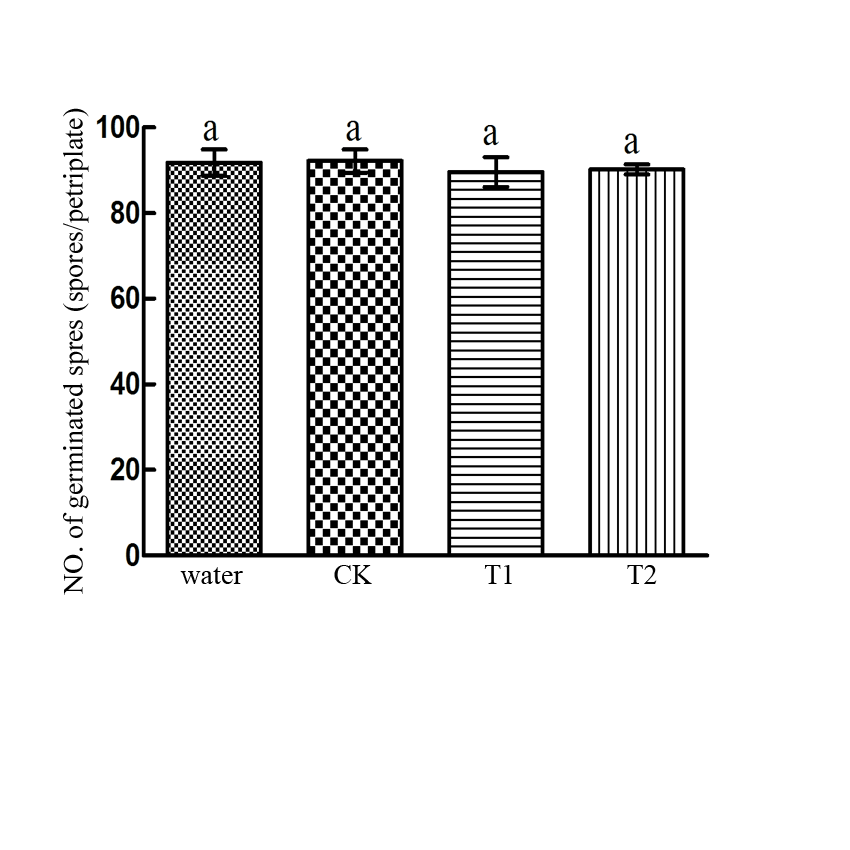


**Figure S1B** The number of germinated spores in concentration of wheat straw decomposing for 0.25 g/ml

**Table S1** The colony diameter in different concentration of wheat straw decomposing in cultivate for 3 days and 6 days

| Concentration | Colony diameter (cm) for 3 day | | | | Colony diameter (cm) for 6 day | | | |
| --- | --- | --- | --- | --- | --- | --- | --- | --- |
| (g/ml) | water | CK | T1 | T2 | water | CK | T1 | T2 |
| 0.10 | 4.88±0.16a | 4.72±0.21ab | 4.58±0.24ab | 4.75±0.31ab | 7.16±0.83a | 6.88±0.38a | 6.98±0.73a | 6.67±0.24a |
| 0.25 | 4.78±0.15a | 4.75±0.11a | 4.68±0.24ab | 4.75±0.28a | 7.04±0.56ab | 6.98±0.63ab | 7.12±0.34a | 7.23±0.43a |
| 0.50 | 4.88±0.23a | 4.78±0.15a | 4.58±0.31ab | 4.59±0.31ab | 7.12±0.43a | 7.01±0.54a | 6.98±0.42ab | 6.89±0.32ab |
