# Supplementary material for: Wheat straw increases the defense response and resistance of watermelon monoculture to Fusarium wilt
Source: BMC Plant Biol. 2019 Dec 11;19:551. doi: 10.1186/s12870-019-2134-y (PMC6907359; doi:10.1186/s12870-019-2134-y)
Supplement: Supplementary file 2 — Additional file 2. Classification of raw reads. [file 12870_2019_2134_MOESM2_ESM.doc]

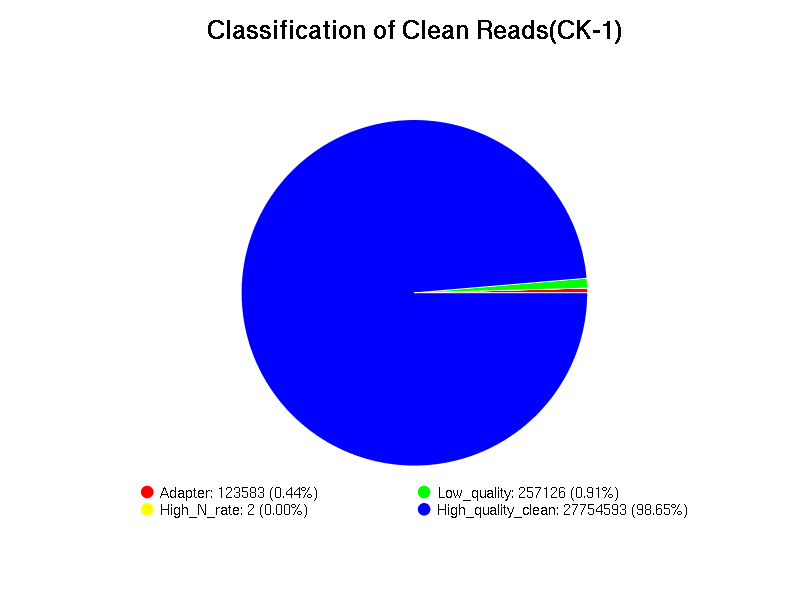

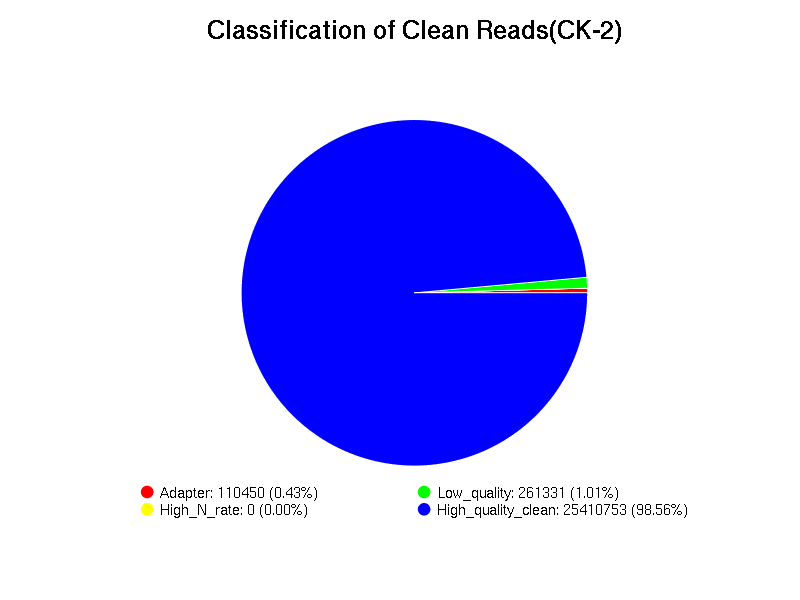

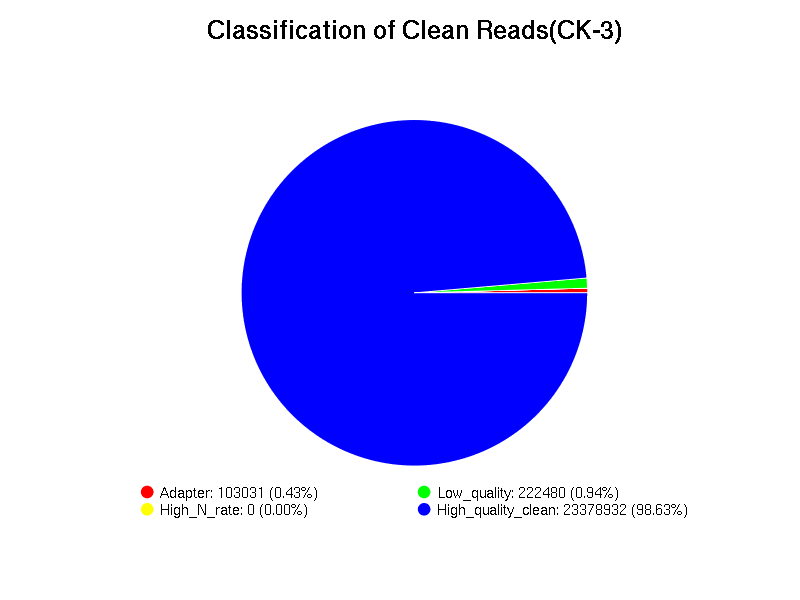

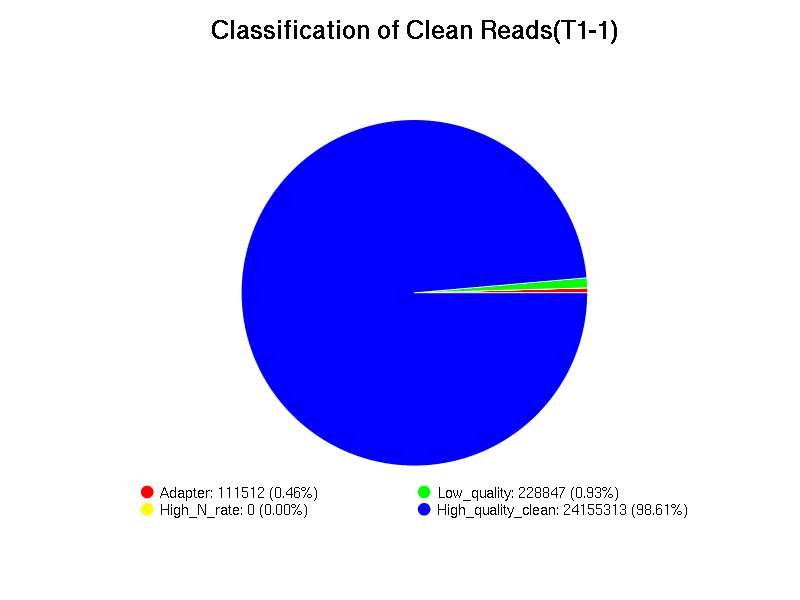


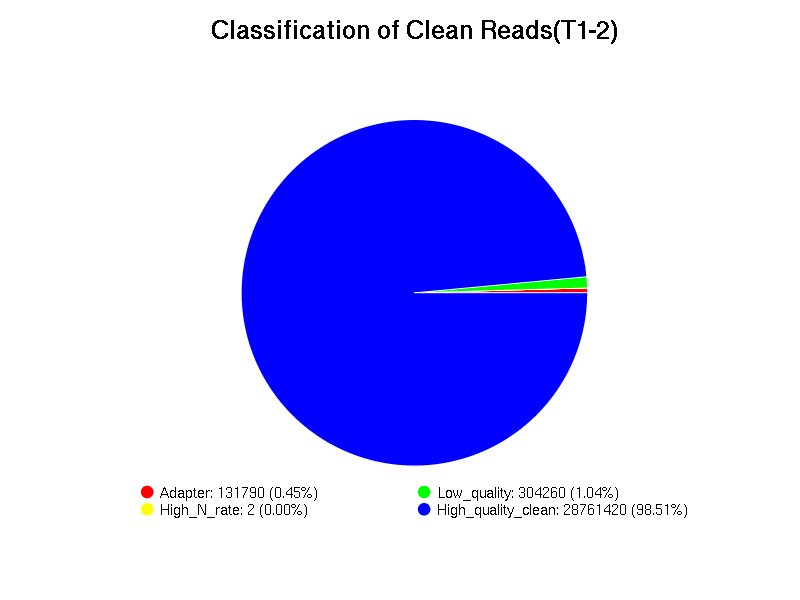

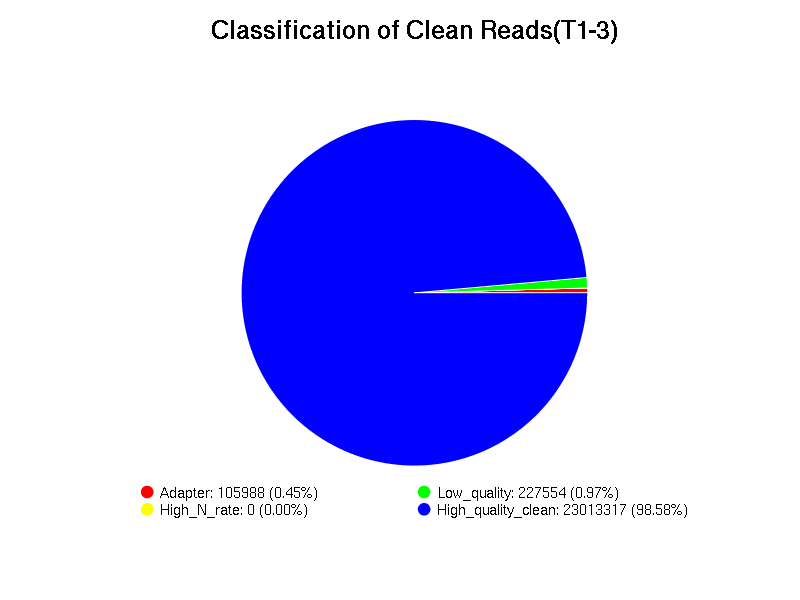


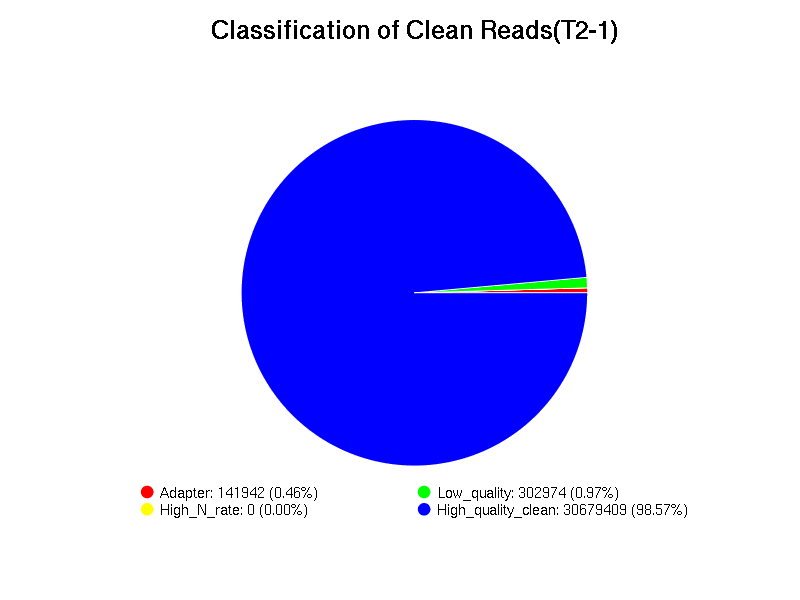

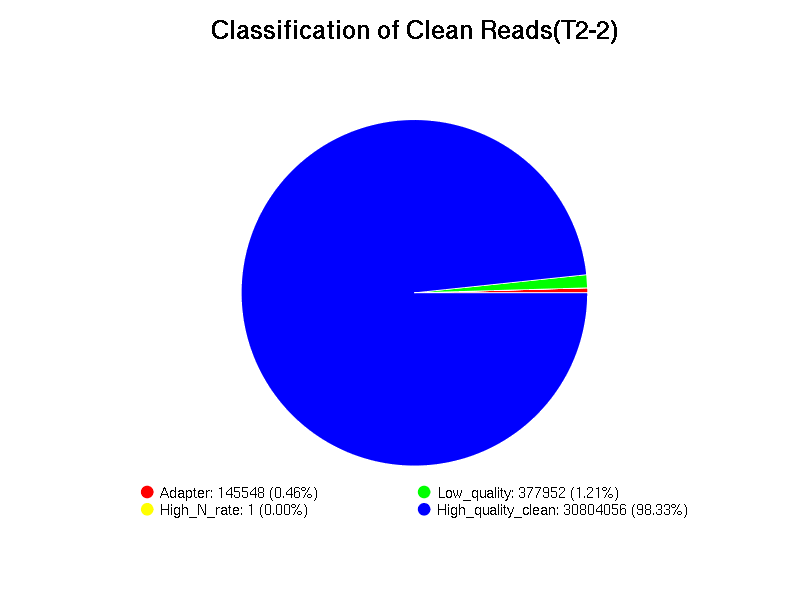


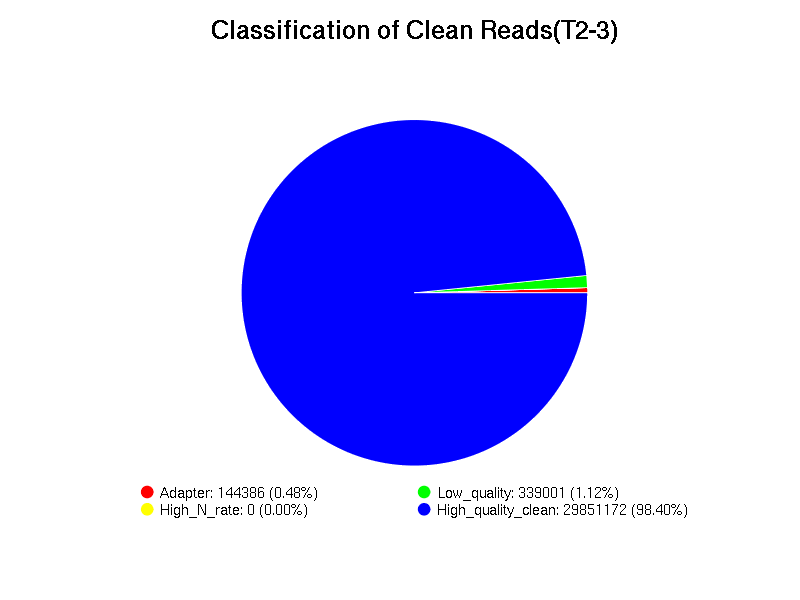


**Figure S2 Classification of raw reads**

CK, without adding wheat straw; T1, adding 1% wheat straw; T2, adding 2% wheat straw
