# Supplementary material for: Wheat straw increases the defense response and resistance of watermelon monoculture to Fusarium wilt
Source: BMC Plant Biol. 2019 Dec 11;19:551. doi: 10.1186/s12870-019-2134-y (PMC6907359; doi:10.1186/s12870-019-2134-y)
Supplement: Supplementary file 3 — Additional file 3. Primers used for qRT-PCR. [file 12870_2019_2134_MOESM3_ESM.doc]

| Primer name | Forward primer sequence (5’-3’) | Reverse primer sequence (5’-3’) |
| --- | --- | --- |
| Cla008727 | AGTATGAACAAAGGGACGGATAG | GGTGCCTCGTAAAGGAAGG |
| Cla013420 | CAAACTTAGGGCTCTGAACG | CTTTGGATTTCTGGGTGGT |
| Cla017226 | CGCCGGACCTTGAGAAG | GGGTTTCATCGGAAATGG |
| Cla009995 | GCGGGCTCATTTGTCCT | CCGATTCCCAGATTCCA |
| Cla006446 | GGTGGCATGTGGGAGCT | GAGGAATGCTGGGAGGTG |
| Cla010664 | CGCAGCAGGGCAAGGTA | GGGTTGTGAGCCAGCATT |
| Cla017432 | ACCTGATTTGGGCGGATTA | CGAGCCTTGTGATGTTGTTG |
| Cla016012 | GTTCTTGACGAGATGGTGGCT | TCCCGATAATACCTAACATACTTGC |
| Cla012342 | GACCATAGATCCGAGAACCG | CCTGCGTAGTACCGTAGCG |
| Cla019382 | TAAAGAGGCAGTGGAAAGG | TGTTCTACCAACGCTATGAG |
| Cla014249 | CATTGGGAAGGAAGGACTC | TTTGCCTGAAGCTGGTG |
| Cla001500 | AAATCGTCAAACGCTGCTCAA | GGCGGAGGAGGCACTGAAA |
| Cla012536 | AGAGAAATCAGGCTCTGCGG | CACAATTCCCTTACCGGCCA |
| Cla007982 | GCCAGCAGACTGACACTTCT | CAGTCGACTCTTCCTGGCTG |
| Cla020086 | TGCTGCAAAGGGCTTAGAGT | GGGCCTAATTTTGCCAAGCC |
| Cla004017 | CCCCATTGATGGTGAACCCA | CGAGTCTCTTCATGGCGGAA |
| Cla016325 | TCGCGTCCAACAAAGGAATG | TACGCTGATCGCCTTGAAGT |
| actin | ACCAACAGTCCGCTTTGTGT | ATTGGGCTCCACTGATTTTG |

**Table S2** Primers used for qRT-PCR
