# Supplementary material for: Wheat straw increases the defense response and resistance of watermelon monoculture to Fusarium wilt
Source: BMC Plant Biol. 2019 Dec 11;19:551. doi: 10.1186/s12870-019-2134-y (PMC6907359; doi:10.1186/s12870-019-2134-y)
Supplement: Supplementary file 4 — Additional file 4. Verification of RNA-Seq data. [file 12870_2019_2134_MOESM4_ESM.doc]

Figure S3


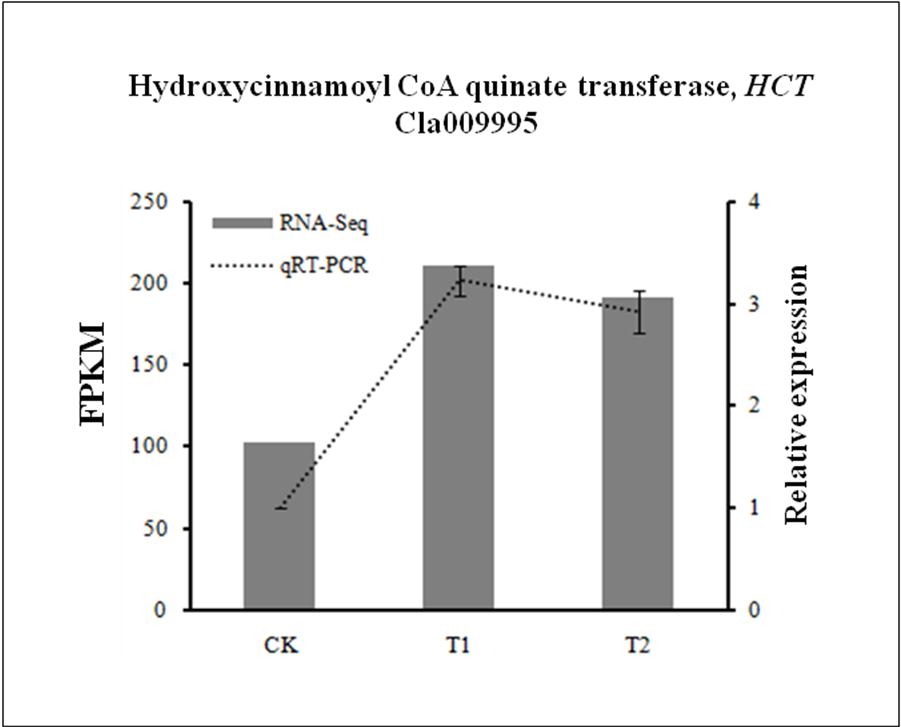

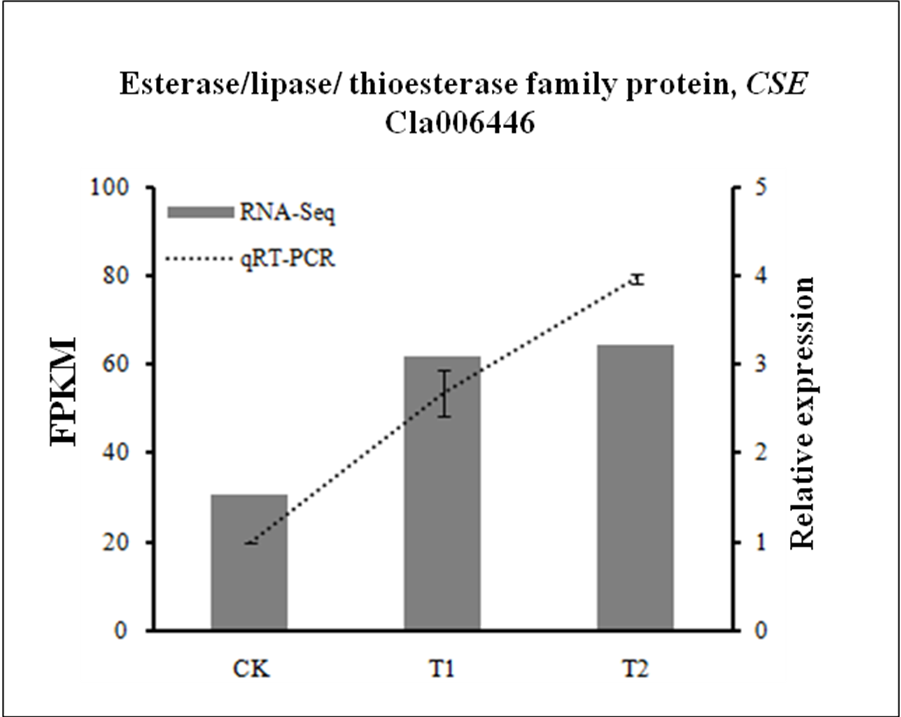


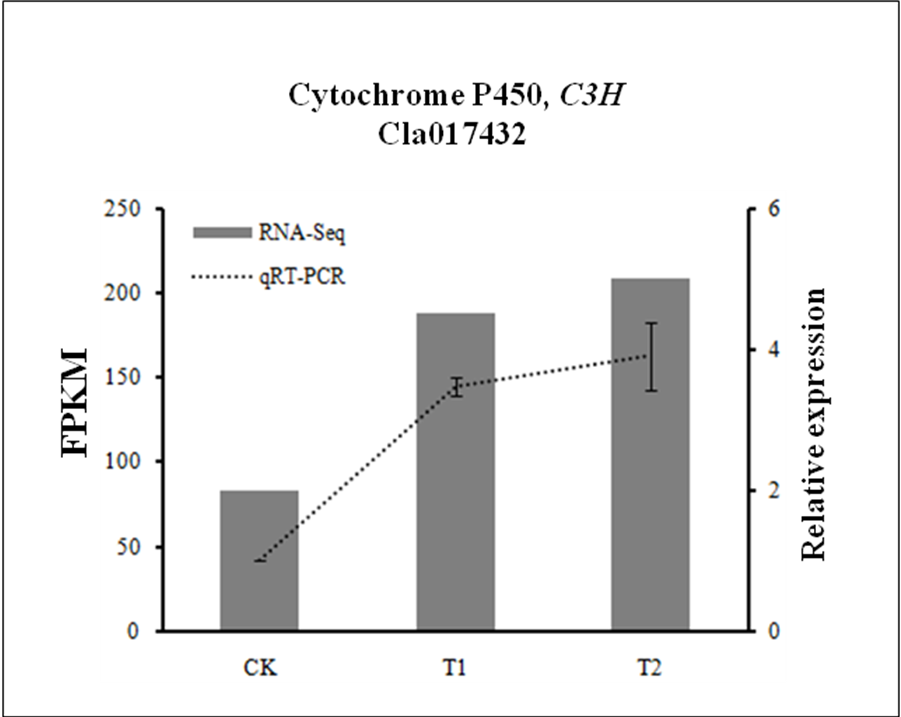

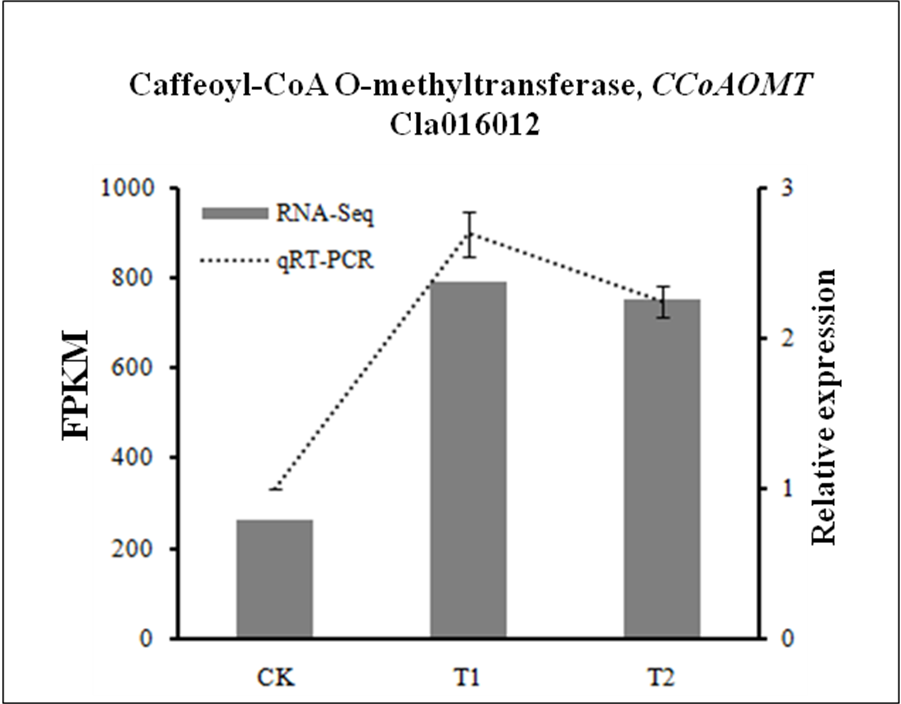


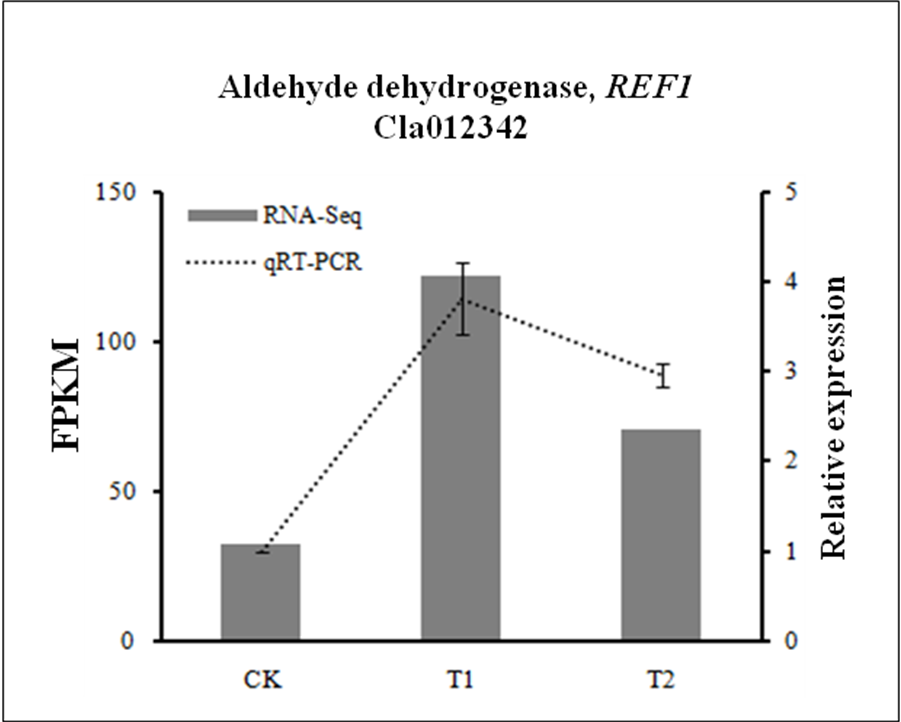

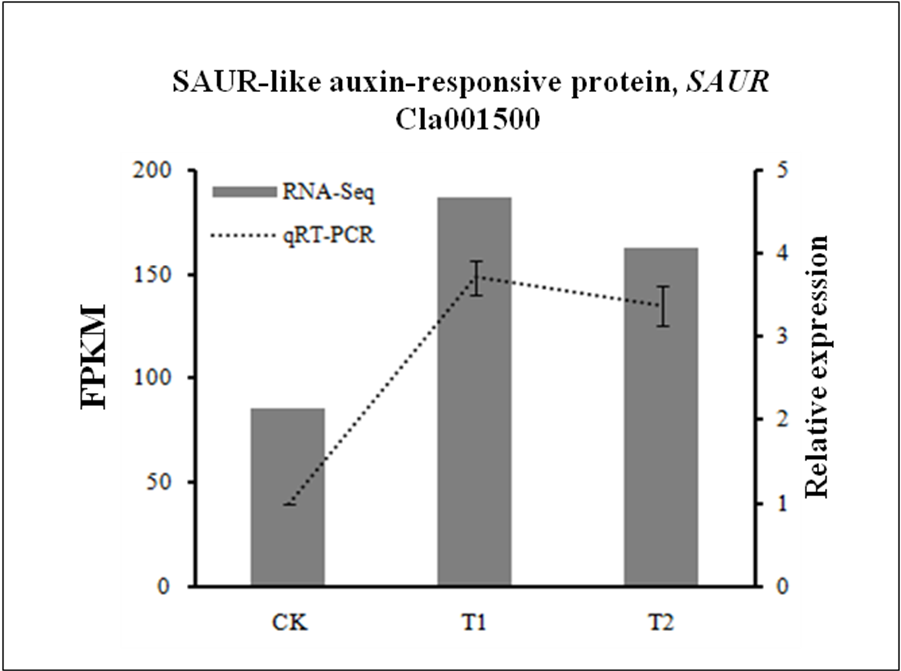


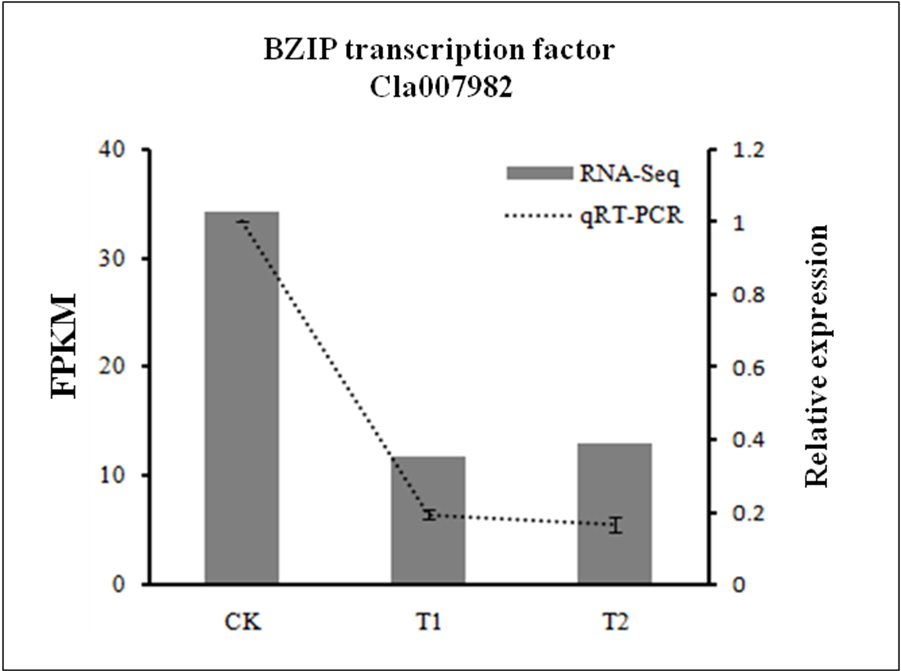

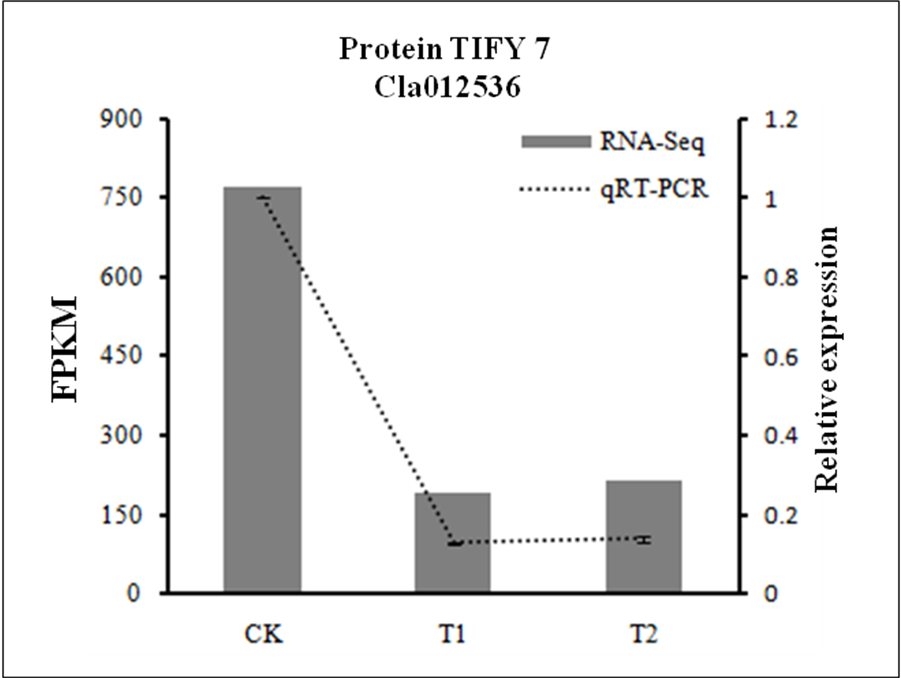


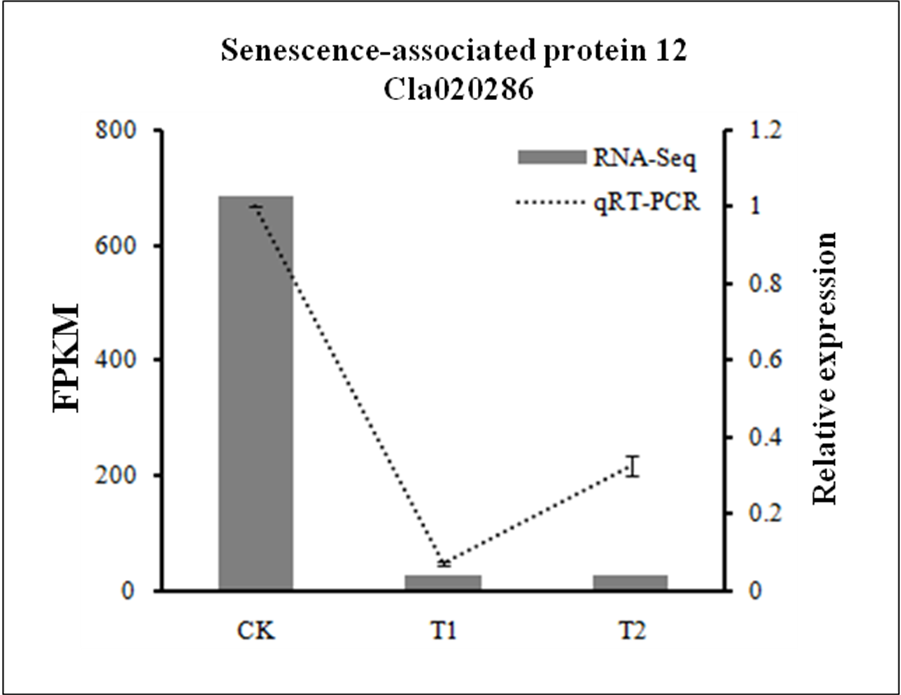

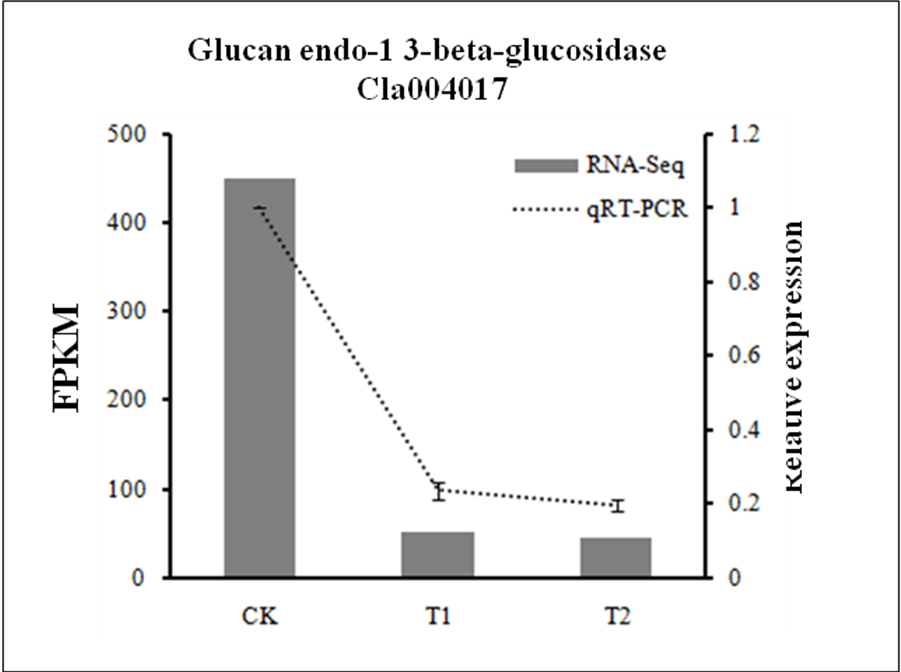


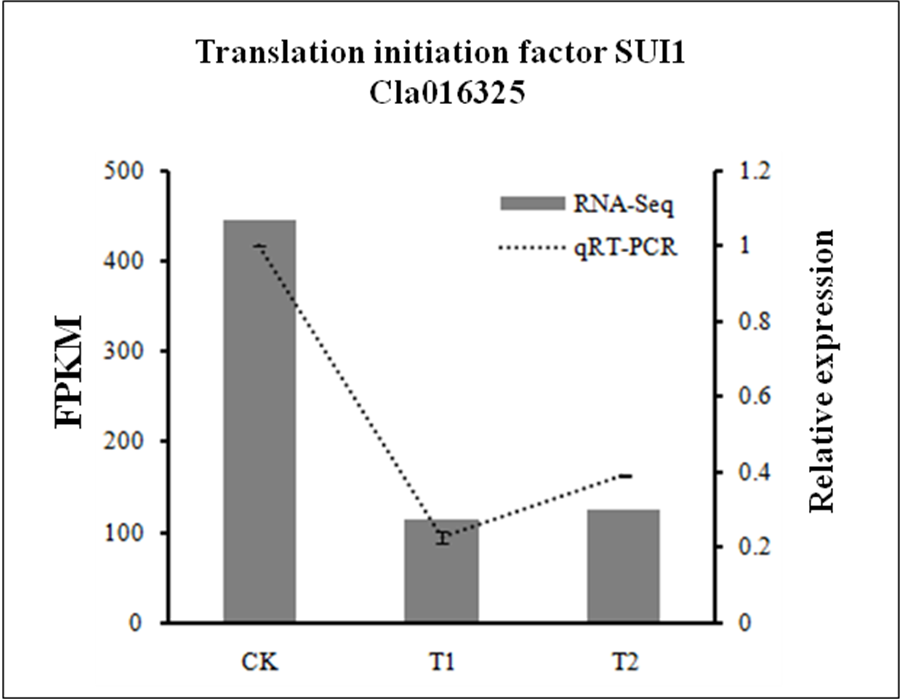


**Figure S3 Veriﬁcation of the RNA-Seq.** The principal Y-axis shows the RNA-Seq data, and the secondary Y-axis shows the relative gene expression levels per qRT-PCR analysis. The bars represent the SEs (n = 3), and the X-axis shows the different treatments.
