# Supplementary material for: Wheat straw increases the defense response and resistance of watermelon monoculture to Fusarium wilt
Source: BMC Plant Biol. 2019 Dec 11;19:551. doi: 10.1186/s12870-019-2134-y (PMC6907359; doi:10.1186/s12870-019-2134-y)
Supplement: Supplementary file 9 — Additional file 9. Heat map diagram of the expression levels of DEGs annotated in hormone signal transduction pathways by KEGG analysis [file 12870_2019_2134_MOESM9_ESM.doc]

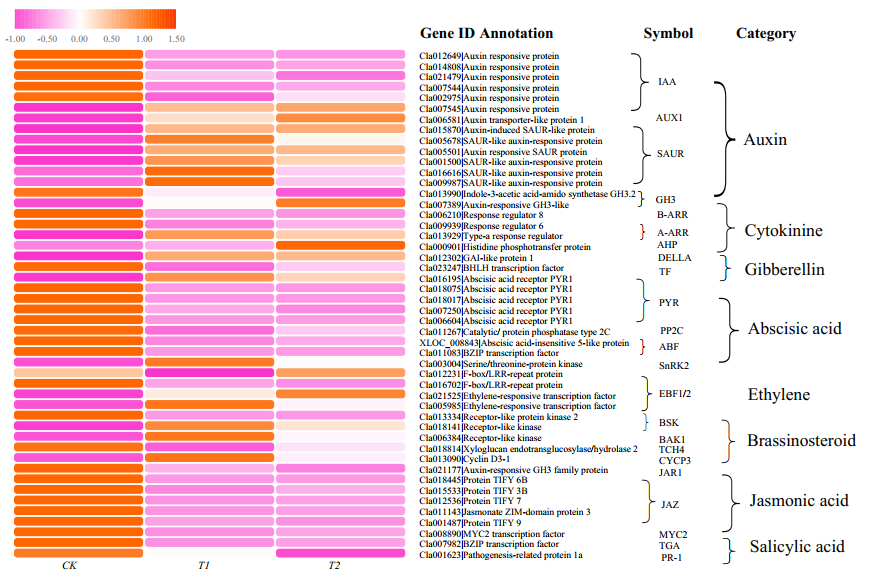


**Figure S6 Heat map diagram of expression levels for DEGs annotated in the hormone signal transduction pathways analyzed by KEGG.** The data for the gene expression levels were normalized to z-scores. CK, without wheat straw; T1, addition 1% wheat straw; T2, addition 2% wheat straw.
