# Supplementary material for: Wheat straw increases the defense response and resistance of watermelon monoculture to Fusarium wilt
Source: BMC Plant Biol. 2019 Dec 11;19:551. doi: 10.1186/s12870-019-2134-y (PMC6907359; doi:10.1186/s12870-019-2134-y)
Supplement: Supplementary file 10 — Additional file 10. PCR primers SAUR genes. [file 12870_2019_2134_MOESM10_ESM.doc]

**Table S6** PCR primers SAUR genes

| Gene ID | Forward primer (5’-3’) | Reverse primer (5’-3’) |
| --- | --- | --- |
| Cla001500 | CGGAAGGATGTTTCACG | GGCGCTTGGCAGTTGTAT |
| Cla015870 | AAATCGTCAAACGCTGCTCAA | GGCGGAGGAGGCACTGAAA |
| Cla005501 | AGAGACATCTTGTGCCCGTG | CCATTATGCCACAGCGACG |
| Cla005678 | GTACTGTCTTCTCCGCTCCG | CGACGAAGCGTTCCATCTCT |
| actin | ACCAACAGTCCGCTTTGTGT | ATTGGGCTCCACTGATTTTG |
